# GVCHAP: A Computing Pipeline for Genomic Prediction and Variance Component Estimation Using Haplotypes and SNP Markers

**DOI:** 10.3389/fgene.2020.00282

**Published:** 2020-04-07

**Authors:** Dzianis Prakapenka, Chunkao Wang, Zuoxiang Liang, Cheng Bian, Cheng Tan, Yang Da

**Affiliations:** ^1^Department of Animal Science, University of Minnesota, Saint Paul, MN, United States; ^2^State Key Laboratory for Agrobiotechnology, China Agricultural University, Beijing, China; ^3^National Engineering Research Center for Breeding Swine Industry, South China Agricultural University, Guangzhou, China

**Keywords:** genomic selection, haplotype, SNP, heritability, prediction accuracy

## Abstract

Haplotype prediction models open many possibilities to improve the accuracy of genomic selection but require more data processing and computing time than single-SNP prediction models. To facilitate haplotype analysis for genomic prediction and estimation using structural and functional genomic information, we developed a computing pipeline to implement haplotype analysis with capabilities for preparation of input data for haplotype analysis, genomic prediction and estimation using GVCHAP, and analysis of GVCHAP results. Data preparation includes utility programs for haplotype imputing; defining haplotype blocks by a fixed number of SNPs, a fixed distance in base pairs per block, or user defined block lengths based on structural or functional genomic information or a mixture of both types of information; and defining haplotype genotypes within each haplotype block. GVCHAP is the main program for genomic prediction and estimation, calculates GREML (genomic restricted maximum likelihood) estimates of variance components and heritabilities, and calculates GBLUP (genomic best linear unbiased prediction) for additive and dominance values of single SNPs as well as additive values of haplotypes with reliability estimates for training and validation populations. A two-step strategy and a method of multi-node processing are implemented to remove the computing bottleneck due to the creation of genomic relationship matrices for large samples. The analysis of GVCHAP results includes calculation of observed prediction accuracies from validation studies and preparation of input files for graphical visualization of heritability estimates of haplotype blocks as well as estimates of SNP effects and heritabilities. The entire pipeline provides an efficient and versatile computing tool for identifying the most accurate haplotype model among many candidate haplotype models utilizing structural and functional genomic information for genomic selection.

## Introduction

Current methods using single nucleotide polymorphism (SNP) markers for genomic evaluation mostly use single-SNP prediction models. Haplotype analysis opens many possibilities of using structural and functional genomic information to improve the accuracy of genomic evaluation and gene discovery (Da, 2015). Compared to studies using single-SNP prediction models, only limited studies were available for using haplotype models in genomic evaluation (Calus et al., 2008; Villumsen et al., 2009; Mulder et al., 2010; Boichard et al., 2012; Cuyabano et al., 2015; Hess et al., 2017; Jónás et al., 2017; Jiang et al., 2018; Jan et al., 2019). Those studies achieved mixed results from little to substantial improvement in prediction accuracy due to the use of haplotypes relative to single-SNP models, and used haplotype blocking methods that are only a fraction of many possible haplotype models. However, investigating many haplotype models per trait is a computing challenge because haplotype models require considerably more data processing and computing resources than required by single-SNP models. Validation study is a commonly used approach to identify best haplotype models from a large number of candidate models but increases data processing work and computational difficulty. The computing pipeline in this article provides a full-featured computing tool for genomic prediction and variance component estimation using haplotypes with capability to minimize the data processing work, reduce computing difficulty, and conduct haplotype genomic prediction and estimation in an automated fashion.

## Methods

### Mixed Model With SNP and Haplotype Effects

GVCHAP implements a multi-allelic haplotype mixed model that treats each haplotype block as a ‘locus’ and each haplotype within the haplotype block as an ‘allele’ (Da, 2015). The mixed model may include SNP additive and dominance effects and haplotype additive effects, with user flexibility to fit any or all of these three genomic effects. Haplotype dominance effects were coded but disabled in GVCHAP due to the large number of haplotype pairs that may exist in some haplotype blocks. The dominance effect of each haplotype pair requires all three genotypes to define, one heterozygous and two homozygous genotypes of the SNP, but one or both homozygous genotypes may be missing when many haplotypes with small frequencies exist.

Based on the quantitative genetics models of haplotypes and SNPs (Da et al., 2014; Da, 2015), the quantitative genetic model with SNP additive and dominance effects as well as haplotype additive effects is:

$$\text{y}=\text{Xb}+\text{Z}\,\left({\text{W}}_{\alpha }\,\alpha_{o}+{\text{W}}_{\delta }\,\delta_{\text{o}}+{\text{W}}_{\alpha \,h}\,\alpha_{\text{ho}}\right)+\text{e}$$

(1) $$=\text{Xb}+\text{Z}\,\left(\text{a}+\text{d}+{\text{a}}_{\text{h}}\right)+\text{e}$$

where **Z** = N × n incidence matrix allocating phenotypic observations to each individual = identity matrix for one observation per individual (N = n), N = number of observations, n = number of individuals, α_o_ = m×1 column vector of SNP additive effects, m = number of SNPs, **W**_α_ = n × m model matrix of α_*o*_, δ_*o*_ = m×1 column vector for dominance effects of SNP genotypes, **W**_δ_ = n × m model matrix of δ_*o*_, α_h_ = n_α*h*_×1 column vector of haplotype additive effects, n_α*h*_ = number of haplotype additive effects, **W**_α*h*_ = n ×n_α*h*_ model matrix of α_h_, **b** = c×1 column vector of fixed effects such as herd-year-season in dairy cattle, c = number of fixed effects, **X** = N×c model matrix of **b**, **a** = **W**_α_α_o_ = SNP genomic additive values, **d** = **W**_δ_δ_o_ = SNP genomic dominance values, **a**_h_ = **W**_α*h*_α_oh_ = haplotype genomic additive values. The haplotype coding represented by ${\text{w}}_{\alpha \,h}^{\mathrm{ij},k}$ in **W**_α*h*_is: ${\text{w}}_{\alpha \,h}^{\mathrm{ij},k}=2\,p_{\text{k}}$ for i,j≠k (a_ij_ and α_1k_ do not share allele k), ${\text{w}}_{\alpha \,h}^{\mathrm{ij},k}=-\left(1-2\,p_{\text{k}}\right)$ for i≠j but i = k or j = k (a_ij_ and α_1k_ share allele k, i≠j), and ${\text{w}}_{\alpha \,h}^{\mathrm{ij},k}=-2\,\left(1-{\text{p}}_{\text{k}}\right)$ for i = j = k (a_ij_ and α_1k_ share allele k, i = j), where a_ij_ = additive value of haplotype genotype with the i^th^ and j^th^ haplotypes, and α_1k_ = additive effect or the average effect of gene substitution as the difference between the allelic (haplotype) effects of the first and the *k*^th^ haplotypes (Da, 2015). SNP codings in **W**_α_ and **W**_δ_ are the same as defined by the single-SNP quantitative genetics model (Da et al., 2014). The first and second moments of Eq. 1 are E(**y**) = **Xb**, and

$$\mathrm{Var}\,\left(y\right)=\text{V}=\text{Z}\,\left(\sigma_{\alpha \,o}^{2}\,{\text{W}}_{\alpha }\,{\text{W}}_{\alpha }^{\prime }+\sigma_{\delta \,o}^{2}\,{\text{W}}_{\delta }\,{\text{W}}_{\delta }^{\prime }+\sigma_{\alpha \,o\,h}^{2}\,{\text{W}}_{\alpha \,h}\,{\text{W}}_{\alpha \,h}^{\prime }\right)\,Z^{\prime }$$

(2) $$+\sigma_{\text{e}}^{2}\,{\text{I}}_{\text{N}}=\text{Z}\,\left({\text{G}}_{\text{a}}+{\text{G}}_{\text{d}}+{\text{G}}_{\text{ah}}\right)\,Z^{\prime }+\sigma_{\text{e}}^{2}\,{\text{I}}_{\text{N}}$$

where $\sigma_{\alpha \,o}^{2}$ = SNP additive variance, $\sigma_{\delta \,o}^{2}$ = SNP dominance variance, $\sigma_{\alpha \,o\,h}^{2}$ = haplotype additive variance, $\sigma_{\text{e}}^{2}$ = residual variance, ${\text{G}}_{\text{a}}=\text{var}\,\left(a\right)=\sigma_{\alpha \,o}^{2}\,{\text{W}}_{\alpha }\,{\text{W}}_{\alpha }^{\prime }$, ${\text{G}}_{\text{d}}=\text{var}\,\left(d\right)=\sigma_{\delta \,o}^{2}\,{\text{W}}_{\delta }\,{\text{W}}_{\delta }^{\prime }$, and ${\text{G}}_{\text{ah}}=\text{var}\left(a\right)=\sigma_{\alpha \,o\,h}^{2}{\text{W}}_{\alpha \,h}{\text{W}}_{\alpha \,h}{}^{\prime }$.

Based on Eqs. 1, 2, the mixed model with genomic relationship matrices is a reparameterized and an equivalent model of Eqs. 1, 2, i.e.,

$$\text{y}=\text{Xb}+\text{Z}\,\left({\text{T}}_{\alpha }\,\alpha +{\text{T}}_{\delta }\,\delta +{\text{T}}_{\alpha \,h}\,\alpha_{\text{h}}\right)+\text{e}$$

(3) $$=\text{Xb}+\text{Z}\,\left(\text{a}+\text{d}+{\text{a}}_{\text{h}}\right)+\text{e}$$

(4) $$\text{Var}\,\left(a\right)=\sigma_{\alpha }^{2}\,{\text{A}}_{g}=\sigma_{\alpha }^{2}\,{\text{T}}_{\alpha }\,{\text{T}}_{\alpha }^{\prime }=\sigma_{\alpha \,o}^{2}\,{\text{W}}_{\alpha }\,{\text{W}}_{\alpha }^{\prime }={\text{G}}_{\text{a}}$$

(5) Var(d)=σδ2Dg=σδ2TδTδ=′σδ⁢o2WδWδ′=Gd

(6) $$\text{Var}\,\left({\text{a}}_{\text{h}}\right)=\sigma_{\alpha \,h}^{2}\,{\text{A}}_{\alpha \,\text{h}}={\text{T}}_{\alpha \,h}\,{\text{T}}_{\alpha \,h}^{\prime }=\sigma_{\alpha \,o\,h}^{2}\,{\text{W}}_{\alpha \,h}\,{\text{W}}_{\alpha \,h}^{\prime }={\text{G}}_{\text{ah}}$$

$$\text{Var}\,\left(y\right)=\text{V}=\text{Z}\,\left(\sigma_{\alpha }^{2}\,{\text{A}}_{\text{g}}+\sigma_{\delta }^{2}\,{\text{D}}_{\text{g}}+\sigma_{\alpha \,h}^{2}\,{\text{A}}_{\text{gh}}\right)\,{\text{Z}}^{\prime }+\sigma_{\text{e}}^{2}\,{\text{I}}_{\text{N}}$$

(7) $$=\text{Z}\,\left({\text{G}}_{\text{a}}+{\text{G}}_{\text{d}}+{\text{G}}_{\text{ah}}\right)\,{\text{Z}}^{\prime }+\sigma_{\text{e}}^{2}\,{\text{I}}_{\text{N}}$$

where **A**_g_ = SNP genomic additive relationship matrix, **D**_g_ = SNP genomic dominance relationship matrix, **A**_gh_ = haplotype genomic additive relationship matrix, ${\text{T}}_{\alpha }={\text{W}}_{\alpha }/{\text{k}}_{\alpha }^{1/2}$, ${\text{T}}_{\delta }={\text{W}}_{\delta }/{\text{k}}_{\delta }^{1/2}$, ${\text{T}}_{\alpha \,h}={\text{W}}_{\alpha \,h}/{\text{k}}_{\alpha \,h}^{1/2}$, **a** = **T**_α_α = **W**_α_α_o_ = SNP genomic additive values, **d** = **T**_δ_δ = **W**_δ_δ_o_ = SNP genomic dominance values, **a**_h_ = **T**_α*h*_α_h_ = **W**_α*h*_α_oh_ = haplotype genomic additive values, $\sigma_{\alpha }^{2}$ = SNP additive variance, $\sigma_{\delta }^{2}$ = SNP dominance variance, $\sigma_{\alpha \,h}^{2}$ = haplotype additive variance, $\sigma_{\text{e}}^{2}$ = residual variance, **V** = phenotypic variance-covariance matrix, and

(8) $$k_{\alpha }=\mathrm{tr}\,\left({\text{W}}_{\alpha }\,{\text{W}}_{\alpha }^{\prime }\right)/n$$

(9) $$k_{\delta }=\mathrm{tr}\,\left({\text{W}}_{\delta }\,{\text{W}}_{\delta }^{\prime }\right)/n$$

(10) $$k_{\alpha \,h}=\mathrm{tr}\,\left({\text{W}}_{\alpha \,h}\,{\text{W}}_{\alpha \,h}^{\prime }\right)/n$$

Each of k_α_, k_δ_ and k_αh_ defined by Eqs. 8–10 is an average of the diagonal elements of ${\text{W}}_{\text{j}}\,{\text{W}}_{\text{j}}^{\prime }$ (j = α, δ, αh). With this definition, variance components $\sigma_{\alpha }^{2}$, $\sigma_{\delta }^{2}$ and $\sigma_{\alpha \,h}^{2}$ can be interpreted as the average of the corresponding variances of all individuals under the original quantitative genetics model of Eqs. 1, 2, i.e.,

(11) $$\sigma_{\alpha }^{2}=t\,r\,\left({\text{G}}_{\text{a}}\right)/n=\sigma_{\alpha \,o}^{2}\,t\,r\,\left({\text{W}}_{\alpha }\,{\text{W}}_{\alpha }^{\prime }\right)/n=k_{\alpha }\,\sigma_{\alpha \,o}^{2}$$

(12) $$\sigma_{\delta }^{2}=\text{tr}\,\left({\text{G}}_{\text{d}}\right)/n=\sigma_{\delta \,o}^{2}\,\text{tr}\,\left({\text{W}}_{\delta }\,{\text{W}}_{\delta }^{\prime }\right)/n={\text{k}}_{\delta }\,\sigma_{\delta \,o}^{2}$$

(13) $$\sigma_{\alpha \,h}^{2}=t\,r\,\left({\text{G}}_{\text{ah}}\right)/n=\sigma_{\alpha \,o\,h}^{2}\,t\,r\,\left({\text{W}}_{\alpha \,h}\,{\text{W}}_{\alpha \,h}^{\prime }\right)/n=k_{\alpha \,h}\,\sigma_{\alpha \,o\,h}^{2}$$

and the genomic relationship matrices in Eqs. 4–6 can be expressed as:

(14) $${\text{A}}_{\text{g}}={\text{T}}_{\alpha }\,{\text{T}}_{\alpha }^{\prime }={\text{W}}_{\alpha }\,{\text{W}}_{\alpha }^{\prime }/{\text{k}}_{\alpha }\,\left(\text{Hayes and Goddard, 2010}\right)$$

(15) Dg=TδTδ=′WδWδ′/kδ

(Da et al., 2014;Wang and Da, 2014)

(16) $${\text{A}}_{\text{gh}}={\text{T}}_{\alpha \,h}\,{\text{T}}_{\alpha \,h}^{\prime }={\text{W}}_{\alpha \,h}\,{\text{W}}_{\alpha \,h}^{\prime }/{\text{k}}_{\alpha \,h}\,\left(\text{Da, 2015}\right)$$

GVCHAP chose to implement the genomic relationship matrices using the average of the diagonal elements in ${\text{W}}_{j}\,{\text{W}}_{\text{j}}^{\prime }$ (j = α, δ, αh) as the denominator, i.e., k_j_ = $\mathrm{tr}\,\left({\text{W}}_{\text{j}}\,{\text{W}}_{\text{j}}^{\prime }\right)/n$ as the denominator (Eqs. 8–10), because this approach yields variance and heritability estimates in the study population that can be a random or an inbred population (Da, 2019). The genomic relationship matrix that uses the total SNP heterozygosity as the denominator (VanRaden, 2008) is not implemented by the current version of GVCHAP, unlike GVCBLUP that implements both methods with VanRaden’s method as Definition I, and the Hayes-Goddard method as Definition II (Wang et al., 2014b). VanRaden’s method preserves the properties of pedigree additive relationships, i.e., a_ii_ = 1 + f and a_ij_ = 2f_ij_, where a_ij_ = additive relationship between the i^th^ and j^th^ individuals, f_ij_ = coancestry coefficient between the i^th^ and j^th^ individuals, and f = inbreeding coefficient; but underestimates genetic variance components and heritability compared to the Hayes-Goddard method when inbreeding is present. The Hayes-Goddard method does not preserve the properties of pedigree additive relationships. Although these two methods for calculating genomic relationship matrices have different interpretations and generally have differences in estimates of variance components and heritabilities, these methods and the quantitative genetics model of Eqs. 1, 2 without using genomic relationships yield identical GBLUP and reliability (Da, 2019). The use of haplotype genomic relationships is in parallel to the use of SNP genomic relationships, but haplotype genomic relationships are not suitable for measuring relationships among individuals such as parent-offspring relationship due to recombination between SNPs within haplotype blocks (Da, 2015). The only practical application of haplotype genomic relationships is for multi-allelic markers such as microsatellite markers but such markers are virtually unused in current genetic research. This was another reason why only one method for defining genomic relationship matrix was implemented in GVCHAP.

### Software Implementation

The GVCHAP program was developed based on the GREML_CE program in the GVCBLUP package for genomic prediction and variance component estimation using SNP markers (Wang et al., 2014b). As in GVCBLUP, GVCHAP is programmed in C++ language using Eigen and Intel Math Kernel library (MKL). Eigen is a C++ template library for linear algebra, supports large dense and sparse matrices. Intel MKL provides BLAS and LAPACK linear algebra routines and is optimized for Intel processors by using shared memory parallel computing technology. The multi-node processing (MNP) program is based on the input section of GVCHAP with modifications for processing the input SNP and haplotype files using multiple nodes. Nearly all the utility programs are written in Python. The GVCHAP implementation of the multi-allelic haplotype model was validated by a R-script that had the same results of EM-REML as GVCHAP for the same testing data. It was widely confirmed that EM-REML and AI-REML converge to the same estimates. The R-script and the testing data are included in the GVCHAP package.

## Results and Discussion

### Structure of GVCHAP Computing Pipeline

**FIGURE 1:**
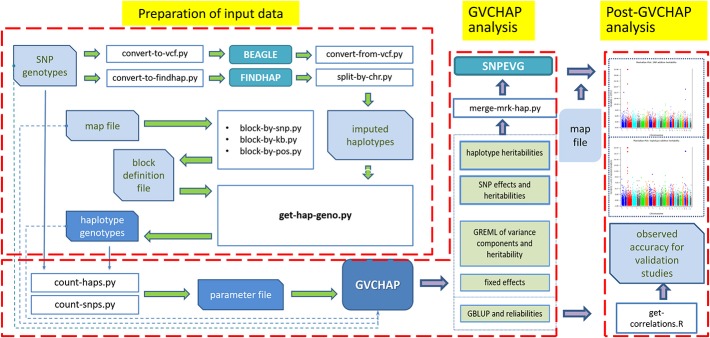
Structure of GVCHAP computing pipeline. The pipeline consists of three components, preparation of input data for haplotype analysis, GVCHAP analysis of genomic prediction and estimation, and post-GVCHAP analysis.

The computing pipeline of GVCHAP (Figure 1) consists of three components: data preparation, GVCHAP analysis, and analysis of GVCHAP results. The complete list of computer programs in this pipeline and the technical details for using these programs are described in the GVCHAP User Manual (Supplementary Material). The following is an overview of this computing pipeline.

### Computing Tools for Preparation of Input Files for GVCHAP Analysis

**FIGURE 2:**
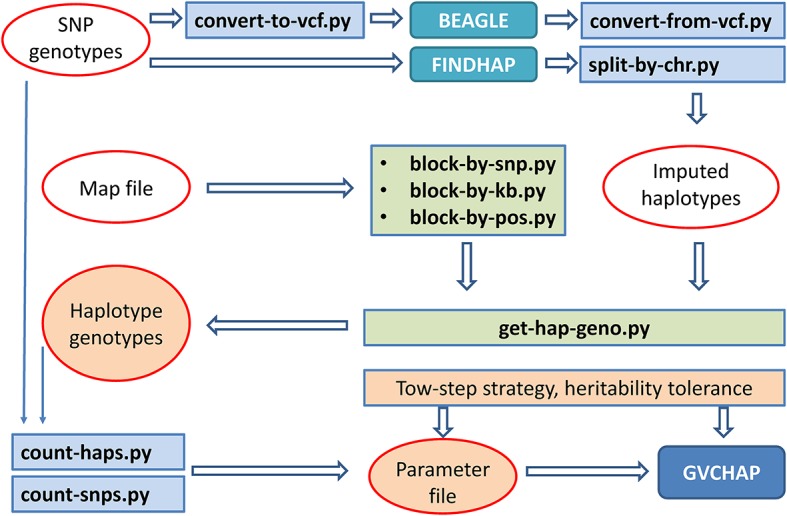
Computing pipeline for preparing input files for GVCHAP analysis.

Five input files are required for running GVCHAP: SNP genotypes, haplotype genotypes, phenotypes, SNP map file, and parameter file. The SNP genotypic files, phenotypic file and map file are provided by the user and remain unchanged for GVCHAP analysis. The haplotype genotypic files can be tedious to prepare, particularly for studies evaluating many haplotype models using validation studies for each model. A set of utility programs prepares the haplotype genotypes and partially fill in the parameter file in an automated fashion. This process starts with converting the format of the user provided SNP genotypic file into the format for BEAGLE (Browning et al., 2018) or FINDHAP (VanRaden et al., 2015), running BEAGLE or FINDHAP to produce imputed haplotypes, dividing the imputed haplotypes into haplotype blocks, and defining haplotype genotypes within each haplotype block (Figure 2).

Dividing haplotypes of each chromosome into haplotype blocks, to be referred to as haplotype blocking, is the first step for defining a specific haplotype model. Three utility programs for haplotype blocking allow three options for defining haplotype blocks: a fixed number of SNPs per block using ‘block-by-snp.py,’ or a fixed distance in kilo-bases per block using ‘block-by-kb.py,’ or a user provided haplotype blocking file that can have various block lengths using ‘block-by-pos.py.’ The user provided blocking file provides flexibility for using various types of structural and functional genomic information such as LD based blocking (LD = linkage disequilibrium) and gene based blocking. The parameter file contains controls for running GVCHAP, including the prediction model, the use of EM-REML and AI-REML, and information about the input and output files. Two utility programs (count-snps.py and count-haps.py) fill in the number of SNPs and the number of haplotype blocks for each SNP genotype file and each haplotype genotype file.

### GVCHAP Analysis

**FIGURE 3:**
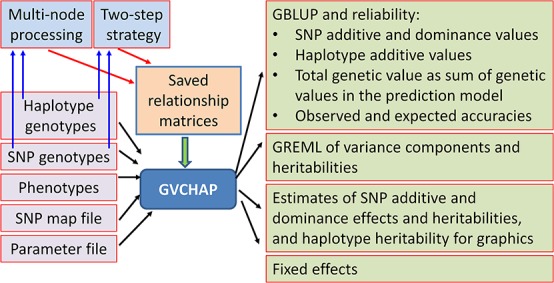
Input and output files of GVCHAP analysis.

The GVCHAP analysis produces GBLUP (genomic best linear unbiased prediction) and GREML (genomic restricted maximum likelihood estimation) for SNP effects and values as well as haplotype additive values with options to improve computing speed (Figure 3).

#### Seven Prediction Models

The prediction model can include SNP additive and dominance values, and haplotype additive values. For these values, GVCHAP offers seven models that include the full model of Eq. 3 and six variations of Eq. 3:

Model 1: SNP additive, dominance and haplotype additive values, **y** = **Xb** + **Z**(**a** + **d** + **a**_h_) + **e**;
Model 2: SNP and haplotype additive values, **y** = **Xb** + **Z**(**a** + **a**_h_) + **e**;
Model 3: SNP dominance values and haplotype additive values, **y** = **Xb** + **Z**(**d** + **a**_h_) + **e**;
Model 4: haplotype additive values only, **y** = **Xb** + **Za**_h_ + **e**;
Model 5: SNP additive and dominance values, **y** = **Xb** + **Z**(**a** + **d**) + **e**;
Model 6: SNP additive values only, **y** = **Xb** + **Za** + **e**;
Model 7: SNP dominance values only, **y** = **Xb** + **Zd** + **e**.

Models 1–4 contain haplotype additive values, and Models 5–7 are SNP models. The comparison between Models 1–4 and Models 5–7 for prediction accuracy provides an estimate whether haplotypes improve the prediction accuracy. For example, haplotypes improved the prediction accuracy if any of the haplotype models (Models 1–4) was more accurate than the three SNP models (Models 5–7) in validation studies. Similarly, validation studies can identify the most accurate model among the seven prediction models. Each of the seven models is configured by the starting values of the variance components in the parameter file through four parameters, var_snp_a, var_snp_d, var_snp_e, and var_hap_a for starting values of SNP additive variance, SNP dominance variance, residual variance, and haplotype additive variance respectively. These seven models and the main results of GBLUP and GREML for each model are summarized in Table 1.

**TABLE 1:** Seven prediction models configured by parameters for starting values of variance components in the parameter file and the main results of GBLUP and GREML for each model.

	**var_snp_a**	**var_snp_d**	**var_snp_e**	**var_hap_a**
Model 1	var_snp_a NPV	var_snp_d NPV	var_snp_e NPV	var_hap_a NPV
	$\hat{\text{a}}$, ${\text{R}}_{\text{a}}^{2}$, ${\hat{\sigma }}_{\alpha }^{2}$, ${\hat{\text{h}}}_{\alpha }^{2}$	$\hat{\text{d}}$, ${\text{R}}_{\text{d}}^{2}$, ${\hat{\sigma }}_{\delta }^{2}$, ${\hat{\text{h}}}_{\delta }^{2}$	${\hat{\sigma }}_{\text{e}}^{2}$	${\hat{\text{a}}}_{\text{h}}$, ${\text{R}}_{\text{ah}}^{2}$, ${\hat{\sigma }}_{\alpha \,h}^{2}$, ${\hat{\text{h}}}_{\alpha \,h}^{2}$, $\hat{\text{g}}$, ${\text{R}}_{\text{g}}^{2}$
Model 2	var_snp_a NPV	#var_snp_d	var_snp_e NPV	var_hap_a NPV
	$\hat{\text{a}}$, ${\text{R}}_{\text{a}}^{2}$, ${\hat{\sigma }}_{\alpha }^{2}$, ${\hat{\text{h}}}_{\alpha }^{2}$		${\hat{\sigma }}_{\text{e}}^{2}$	${\hat{\text{a}}}_{\text{h}}$, ${\text{R}}_{\text{ah}}^{2}$, ${\hat{\sigma }}_{\alpha \,h}^{2}$, ${\hat{\text{h}}}_{\alpha \,h}^{2}$, $\hat{\text{g}}$, ${\text{R}}_{\text{g}}^{2}$
Model 3	#var_snp_a	var_snp_d NPV	var_snp_e NPV	var_hap_a NPV
		$\hat{\text{d}}$, ${\text{R}}_{\text{d}}^{2}$, ${\hat{\sigma }}_{\delta }^{2}$, ${\hat{\text{h}}}_{\delta }^{2}$	${\hat{\sigma }}_{\text{e}}^{2}$	${\hat{\text{a}}}_{\text{h}}$, ${\text{R}}_{\text{ah}}^{2}$, ${\hat{\sigma }}_{\alpha \,h}^{2}$, ${\hat{\text{h}}}_{\alpha \,h}^{2}$, $\hat{\text{g}}$, ${\text{R}}_{\text{g}}^{2}$
Model 4	#var_snp_a	#var_snp_d	var_snp_e NPV	var_hap_a NPV
			${\hat{\sigma }}_{\text{e}}^{2}$	${\hat{\text{a}}}_{\text{h}}$, ${\text{R}}_{\text{ah}}^{2}$, ${\hat{\sigma }}_{\alpha \,h}^{2}$, ${\hat{\text{h}}}_{\alpha \,h}^{2}$
Model 5	var_snp_a NPV	var_snp_d NPV	var_snp_e NPV	#var_hap_a
	$\hat{\text{a}}$, ${\text{R}}_{\text{a}}^{2}$, ${\hat{\sigma }}_{\alpha }^{2}$, ${\hat{\text{h}}}_{\alpha }^{2}$	$\hat{\text{d}}$, ${\text{R}}_{\text{d}}^{2}$, ${\hat{\sigma }}_{\delta }^{2}$, ${\hat{\text{h}}}_{\delta }^{2}$, $\hat{\text{g}}$, ${\text{R}}_{\text{g}}^{2}$	${\hat{\sigma }}_{\text{e}}^{2}$	
Model 6	var_snp_a NPV	#var_snp_d	var_snp_e NPV	#var_hap_a
	$\hat{\text{a}}$, ${\text{R}}_{\text{a}}^{2}$, ${\hat{\sigma }}_{\alpha }^{2}$, ${\hat{\text{h}}}_{\alpha }^{2}$		${\hat{\sigma }}_{\text{e}}^{2}$	
Model 7	#var_snp_a	var_snp_d NPV	var_snp_e NPV	#var_hap_a
		$\hat{\text{d}}$, ${\text{R}}_{\text{d}}^{2}$, ${\hat{\sigma }}_{\delta }^{2}$, ${\hat{\text{h}}}_{\delta }^{2}$	${\hat{\sigma }}_{\text{e}}^{2}$	

*A non-zero positive value (NPV) after the parameter activates the genetic value in the prediction model, and a ‘#’ sign in front of the parameter removes the genetic value from the prediction model. $\hat{\text{a}}$ = GBLUP of SNP additive value. ${\text{R}}_{\text{a}}^{2}$ = reliability of $\hat{\text{a}}$. $\hat{\text{d}}$ = GBLUP of SNP dominance value. ${\text{R}}_{\text{d}}^{2}$ = reliability of $\hat{\text{d}}$. $\hat{\text{g}}$ = GBLUP of total genotypic value, = $\hat{\text{a}}$ + $\hat{\text{d}}$ + ${\hat{\text{a}}}_{\text{h}}$ for Model 1, = $\hat{\text{a}}$ + ${\hat{\text{a}}}_{\text{h}}$ for Model 2, = $\hat{\text{d}}$ + ${\hat{\text{a}}}_{\text{h}}$ for Model 3, = $\hat{\text{a}}$ + $\hat{\text{d}}$ for Model 5. ${\text{R}}_{\text{g}}^{2}$ = reliability of $\hat{\text{g}}$. ${\hat{\sigma }}_{\alpha }^{2}$ = GREML estimate of SNP additive variance. ${\hat{\sigma }}_{\delta }^{2}$ = GREML estimate of SNP dominance variance. ${\hat{\sigma }}_{\text{e}}^{2}$ = GREML estimate of residual variance. ${\hat{\sigma }}_{\alpha \,h}^{2}$ = GREML estimate of haplotype additive variance. ${\hat{\text{h}}}_{\alpha }^{2}$ = GREML estimate of SNP additive heritability. ${\hat{\text{h}}}_{\delta }^{2}$ = GREML estimate of SNP dominance heritability. ${\hat{\text{h}}}_{\alpha \,h}^{2}$ = GREML estimate of haplotype additive heritability.*

### GREML Estimates of Variance Components and Heritabilities

GVCHAP calculates GREML estimates of variance components and hertiabilities for each of the genetic effects in the prediction model using a combination of EM-REML and AI-REML for iterative solutions adopted from GVCBLUP (Wang et al., 2014b). The program starts with a minimum of two EM-REML iterations, switches to AI-REML at iteration 3 by default, and switches back to EM-REML automatically when AI-REML fails. GREML calculates estimates of variance components with tolerance values, and heritability estimates with tolerance values and standard deviations.

GREML estimates may provide helpful information for choosing among the seven prediction models in Table 1. We recommend GREML with all types of genetic values in the prediction model (Model 1) as the initial GVCHAP analysis to determine whether any type of genetic values has a zero or near-zero heritability, and remove such genetic values from the prediction model. The inclusion of genetic values with negligible heritability may cause slow convergence for GREML and may only have negligible contribution to prediction accuracy. Therefore, removing such genetic values from the prediction model may significantly improve the computing speed with negligible change (positive or negative) to prediction accuracy. For example, the appropriate prediction model should be Model 2 if SNP dominance heritability is negligible, Model 3 if SNP additive heritability is negligible, or Model 4 if SNP additive and dominance hertiabilities are both negligible.

### GBLUP, Reliability, and Expected Prediction Accuracy for Predicting Genetic Values

Converged GREML estimates of variance components are used for calculating GBLUP and reliability for each type of genetic values and the sum of SNP and haplotype values. In the output file for GBLUP and reliability, an individual is flagged with ‘T’ if the individual is in the training population with phenotypic observations, or ‘V’ if the individual has missing phenotypic value or is in the validation population with phenotypic value set as missing value. For the example of Model 1 with SNP additive and dominance values as well as haplotype additive values, GBLUP and reliability for each and the sum of these values are calculated. After sorting the output file by training (T) and validation (V) populations, the GBLUP estimates are:

(17) $$\hat{\text{a}}=\left({\hat{\text{a}}}_{1}^{\prime },{\hat{\text{a}}}_{0}^{\prime }\right)=\mathrm{GBLUP}\,\mathrm{of}\,\mathrm{SNP}\,\mathrm{additive}\,\mathrm{values}$$

(18) $$\hat{\text{d}}=\left({\hat{\text{d}}}_{1}^{\prime },{\hat{\text{d}}}_{0}^{\prime }\right)=\mathrm{GBLUP}\,\mathrm{of}\,\mathrm{SNP}\,\mathrm{dominance}\,\mathrm{values}$$

(19) $${\hat{\text{a}}}_{\text{h}}=\left({\hat{\text{a}}}_{\mathrm{h1}}^{\prime },{\hat{\text{a}}}_{\mathrm{h0}}^{\prime }\right)=\mathrm{GBLUP}\,\mathrm{of}\,\mathrm{haplotype}\,\mathrm{additive}\,\mathrm{values}$$

(20) $$\hat{\text{g}}=\hat{\text{a}}+\hat{\text{d}}+{\hat{\text{a}}}_{\text{h}}=\mathrm{GBLUP}\,\mathrm{of}\,\mathrm{total}\,\mathrm{genotypic}\,\mathrm{values}$$

where ‘∧’ indicates estimated value, subscript ‘1’ indicates training population, and subscript ‘0’ indicates validation population or individuals with missing phenotypic observations. Each of the above GBLUP estimates is accompanied by its reliability. The square root of a reliability estimate is the correlation between GBLUP and the unobservable true genetic value being predicted by the GBLUP, and is the expected accuracy for predicting the unobservable true genetic valu’e, **a**, **d**, **a**_h_, or *g* = *a* + *d* + **a**_h_. In the absence of validation studies, reliability or the expected prediction accuracy is the measure of prediction accuracy for a type of genetic value, e.g., SNP additive or dominance value, or haplotype additive value, or the sum of all these genetic values. The reliability formula for $\hat{\text{g}}=\hat{\text{a}}+\hat{\text{d}}+{\hat{\text{a}}}_{\text{h}}$ of Model 1 is:

$${\text{R}}_{\text{gi}}^{2}={\left(\begin{array}{c} {\text{G}}_{\alpha }\,{\text{Z}}^{\prime }\,{\text{PZG}}_{\alpha }+{\text{G}}_{\delta }\,{\text{Z}}^{\prime }\,{\text{PZG}}_{\delta }+{\text{G}}_{\alpha \,h}\,{\text{Z}}^{\prime }\,{\text{PZG}}_{\alpha \,h}\\ +{\text{G}}_{\alpha }\,{\text{Z}}^{\prime }\,{\text{PZG}}_{\delta }+{\text{G}}_{\delta }\,{\text{Z}}^{\prime }\,{\text{PZG}}_{\alpha }+{\text{G}}_{\alpha }\,{\text{Z}}^{\prime }\,{\text{PZG}}_{\alpha \,h}\\ +{\text{G}}_{\alpha \,h}\,{\text{Z}}^{\prime }\,{\text{PZG}}_{\alpha }+{\text{G}}_{\delta }\,{\text{Z}}^{\prime }\,{\text{PZG}}_{\alpha \,h}+{\text{G}}_{\alpha \,h}\,{\text{Z}}^{\prime }\,{\text{PZG}}_{\delta } \end{array}\right)}_{\text{ii}}$$

(21) $$/\left({\text{A}}_{\text{g}}^{\text{ii}}\,\sigma_{\alpha }^{2}+D_{\text{g}}^{\text{ii}}\,\sigma_{\delta }^{2}+A_{\text{gh}}^{\text{ii}}\,\sigma_{\alpha \,h}^{2}\right)$$

where **P** = **V**^−1^−**V**^−1^**X**(**X**′**V**^−1^**X**)−**X**′**V**^−1^; ${\text{A}}_{\text{g}}^{\text{ii}}$, ${\text{D}}_{\text{g}}^{\text{ii}}$ and ${\text{A}}_{\text{gh}}^{\text{ii}}$ are the i^th^ diagonal elements of **A**_g_, **D**_g_ and **a**_gh_ respectively; and subscripts ii of the numerator indicates the i^th^ diagonal element of the numerator matrix. The reliability formula for any of Models 2–7 can be readily derived from Eq. 21, e.g., the reliability of Model 2 is obtained from Eq. 21 by deleting all terms involving ‘δ.’

### Observed and Expected Accuracy for Predicting Phenotypic Values

For a validation study, this computing pipeline has a utility program (get-correlations.R) calculating the observed accuracy for predicting the phenotypic values using a type of genetic values (e.g., SNP additive values or haplotype additive values) or a sum of several types of genetic values (e.g., the sum of SNP dominance values and haplotype additive values) for each validation. This observed accuracy is calculated as the correlation between the predicted genetic values and the phenotypic values in the validation population (individuals flagged as ‘V’ in the output file) that were omitted when calculating GBLUP. The expected accuracy for predicting phenotypic values is the product between the expected accuracy for predicting genetic values and the square root of heritability (Legarra et al., 2008). For genomic prediction using a type of genetic values, e.g., SNP additive or dominance values, or haplotype additive values, or a sum of these genetic values, the observed and expected accuracies for predicting phenotypic values and the expected accuracies for predicting genetic values are:

(22) $${\hat{\text{R}}}_{0\,p\,i}=\text{corr}\,\left({\hat{\text{g}}}_{0\,\text{i}}^{\prime },{\text{y}}_{0}\right)$$

(23) $${\hat{R}}_{0\,p}=\sum_{i=1}^{k}{\hat{R}}_{0\,p\,i}/k$$

(24) $${\text{R}}_{0\,g\,i}=\sum_{j=1}^{{\text{n}}_{0\,\text{i}}}{\text{R}}_{0\,g\,i\,j}/n_{0\,\text{i}}$$

(25) $${\text{R}}_{0\,g}=\sum_{i=1}^{\text{k}}{\text{R}}_{0\,g\,i}/k$$

(26) $${\text{R}}_{0\,p}={\text{R}}_{0\,g}\,\sqrt{{\text{h}}^{2}}$$

where ${\hat{\text{R}}}_{0\,p\,i}$ = observed prediction accuracy for predicting the phenotypic value of the i^th^ validation population, ${\hat{\text{R}}}_{0\,p}$ = observed prediction accuracy for predicting the phenotypic value of k validation populations such as those from a k-fold validation study, R_0gij_ = expected prediction accuracy for predicting the genotypic values of the j^th^ individual in the i^th^ validation population calculated as the square root of reliability from the GVCHAP output file for GBLUP and reliability based on Eq. 21,R_0gi_ = expected prediction accuracy for predicting the genotypic values of the i^th^ validation population, n_0i_ = number of individuals in the validation population, R_0g_ = expected prediction accuracy for predicting the genotypic values of k validation populations, R_0p_ = expected prediction accuracy for predicting the phenotypic values of k validation populations, and h^2^ = heritability. The get-correlations. R program calculates Eqs. 22–25, but Eq. 26 needs to be calculated by the user using results in the GBLUP and GREML output files.

### Haplotype and SNP Heritability Estimates for Graphic Visualization

**FIGURE 4:**
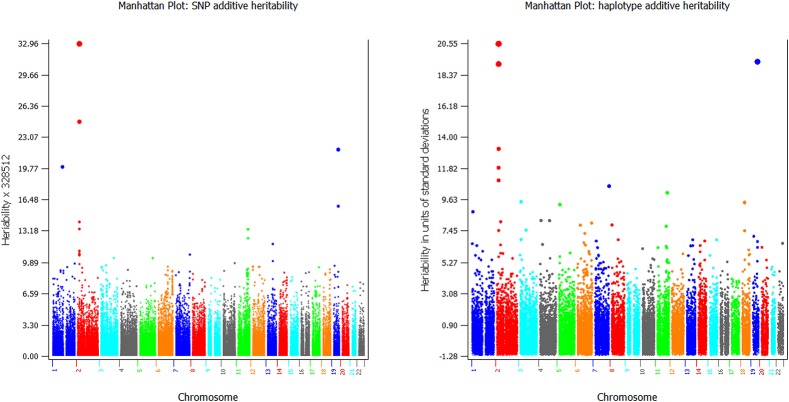
Examples of graphical visualization of SNP heritabilities **(left)** and haplotype heritabilities **(right)**.

GVCHAP calculates and saves SNP effects and heritability estimates, and saves haplotype heritability estimates in separate files for graphical visualization using SNPEVG2 (Wang et al., 2012) to identify SNPs and haplotype blocks with high heritability estimates. The graphical visualization of SNP effects and heritability estimates were available in GVCBLUP (Wang et al., 2014b), and the graphical visualization of haplotype heritability estimates is new to GVCHAP. A utility program (merge-mrk-hap.py) merges the two output files for haplotype heritabilities and SNP effects and heritabilities as a “.snpe” file that SNPEVG2 recognizes as an input file. Manhattan plots of and chromosome graphs of SNP and haplotype heritabilities can be produced by SNPEVG2, as shown by the example of Figure 4. The Manhattan plot and chromosome graphs of haplotype heritabilities may reveal chromosome regions with high heritability estimates that were not observed from the SNP plots and graphs.

### Fixed Effects, and Genomic and Non-genetic Prediction of Phenotypic Values

GVCHAP calculates and saves fixed effects in a separate file and outputs the estimates of the fixed effects for all individuals. Two types of fixed effects can be included in the prediction model, classification variable for a fixed factor with a small number of levels such as male or female, and covariable for a fixed factor with many levels such as age. Selected SNPs can be fitted as fixed effects through declarations in the parameter file for column positions of those SNPs in the phenotype file, typically as covariables to minimize the use of degrees of freedom such that the residual degree of freedom is still sufficiently large.

Three potential applications involve the use of fixed effects. These include estimation of SNP contributions to prediction accuracy and genomic heritability (Tan et al., 2017), genomic prediction using fixed SNP effects for traits with large SNP effects (Spindel et al., 2015), and prediction of phenotypic values using genomic and non-genetic prediction (GNG) that combines genomic prediction and estimates of non-genetic fixed effects such as age and gender. For GNG, the current version of GVCHAP supports samples with one phenotypic observation per individual (N = n), and the general expression of the GNG values under this assumption is:

(27) $$\hat{\text{y}}=\text{X}\,\hat{\text{b}}+\hat{\text{g}}$$

where $\hat{\text{g}}=\hat{\text{a}}+\hat{\text{d}}+{\hat{\text{a}}}_{\text{h}}$ for Model 1, $=\hat{\text{a}}+{\hat{\text{a}}}_{\text{h}}$ for Model 2, $=\hat{\text{d}}+{\hat{\text{a}}}_{\text{h}}$ for Model 3, $={\hat{\text{a}}}_{\text{h}}$ for Model 4, $=\hat{\text{a}}+\hat{\text{d}}$ for Model 5, $=\hat{\text{a}}$ for Model 6, and $=\hat{\text{d}}$ for Model 7.

### Computing Speed of GVCHAP

The computing time of GVCHAP for calculating SNP additive and dominance genomic relationship matrices, haplotype additive relationship matrix, and each GREML iteration was evaluated using five samples and the Mesabi supercomputer at the University of Minnesota (Table 2). The results showed that the creation of the haplotype additive genomic matrix was the most time-consuming and memory-intensive computation, and could require 20 times as much time as creating the SNP additive and dominance genomic relationship matrices. For 15,098 individuals and 657,024 SNPs, the haplotype additive genomic relationship matrix required 22.11 h, whereas the SNP additive and dominance genomic relationship matrices together required 1.32 or 0.66 h per matrix. In contrast, each iteration required little time for any of the four samples, 19–82 s per iteration. To reduce the computing time for matrix creation, we developed two computing strategies, a strategy to remove repeated computations in validation studies and multivariate analysis, and a strategy of multi-node processing to eliminate the computing difficulty for creating the haplotype additive genomic relationship matrix. As the number of SNPs decreases, the computing speed for calculating the genomic matrices increases. For 7549 individuals with 82,128 SNPs, the calculation of the haplotype genomic additive relationship matrix required only 1.31 h, compared to 7.88 h when the number of SNPs became four times as many for the same number of individuals.

**TABLE 2:** Computing time of GVCHAP using the Mesabi supercomputer.

	**GVCHAP using one node with 62 Gb memory**				**Multi-node processing (MNP)**
Number of individuals (n)	7549	7549	15,098	15,098	37,745^a^
Number of SNPs (m)	82,128	328,512	328,512	657,024	328,512
SNP **A**_g_ and **D**_g_	0.12 h	0.71 h	0.70 h	1.32 h	2.67 h
Haplotype **A**_gh_	1.31 h	7.88 h	14.31 h	22.11 h	≈3 h^b^
Time per iteration	19 s^c^	37 s^c^	82 s^c^	62 s^c^	22–29 min^c^ (one node)

*^*a*^Haplotype genotypes of this dataset could not be processed by a single node with 62 Gb memory limit and was used as an example using the MNP method. The haplotype genotypes were divided into 75 files each with 200 blocks for most of the 75 files that were processed using 75 different nodes. ^*b*^Each ${\text{W}}_{\alpha \,h}^{i}\,{\text{W}}_{\alpha \,h}^{i^{\prime }}$ file required 0.831 ± 0.248 h to process, and the summation of the 75 ${\text{W}}_{\alpha \,h}^{i}\,{\text{W}}_{\alpha \,h}^{i^{\prime }}$ matrices and the calculation of ${\text{A}}_{\text{gh}}={\text{W}}_{\alpha \,h}\,{\text{W}}_{\alpha \,h}^{\prime }/{\text{k}}_{\alpha \,h}$ took about 1.66 h to complete. Adding 0.831 and 1.66 h indicates the calculation of **A**_gh_ approximately required 3 h. ^*c*^The computing time per iteration is affected by the number of jobs running on the Mesabi supercomputer.*

### Two-Step Strategy to Remove Repeated Computing for Genomic Relationship Matrices

To remove repeated calculations to create genomic relationship matrices in validation studies and multivariate analysis, we designed a two-step strategy that saves genomic relationship matrices from the first run as binary files and loads the saved genomic relationship matrices for the remaining runs. For the example of a 10-fold validation, genomic relationship matrices are calculated only for the first of the 10 validation runs, and the remaining 9 validation runs load the stored genomic relationship matrices calculated and saved by the first run. This strategy virtually eliminates computing time for creating genomic relationship matrices when running GVCHAP. Our experience showed loading the saved genomic relationship matrices was nearly instantaneous and required only negligible computing times. With this strategy, days of computing time could be saved even for the smallest sample with 7549 individuals and 328,512 SNPs in our test runs (Table 2). For a study investigating many candidate haplotype models for multiple traits using k-fold validations (e.g., 10-fold), days even months of computing time could be saved using this two-step strategy alone (Table 3).

**TABLE 3:** Approximate saving of computing time of GVCHAP due to the two-step strategy or multi-node processing (MNP) for a 10-fold validation study relative to the use of a single node of the Mesabi supercomputer without the two-step strategy of MNP.

Number of individuals (n)	7549	15,098	15,098
Number of SNPs (m)	328,512	328,512	657,024
**Saving in computing time**			
10-fold validation model/trait	3.22 days	6 days	9 days
10-fold validation 10 models per trait	32.2 days	60 days	90 days

### Multi-Node Processing (MNP) for Genomic Relationship Matrices of Large Samples

The long computing time required by GVCHAP to create a haplotype additive genomic relationship matrix using a single node (Table 2) was due to the successive processing of haplotypes by chromosome. This successive processing results in the waiting of the next chromosome to be processed until the current chromosome finished its processing, which involves reading the haplotype genotype data, calculation of allele (haplotype) frequencies, and matrix multiplication and addition. Moreover, the memory limit of a single node sets a limit for the sample size and number of haplotypes that can be processed. To remove the limitation of using successive processing of the haplotype genotypes and a single node, we developed a multi-node processing (MNP) approach that divides the haplotype genotype files into s small files. One node processes each of the s files and all the s small files are processed simultaneously using different nodes. Although SNP additive and dominance genomic relationship matrices only required minor computing time relative to the time required by haplotypes (Table 2), the MNP approach is also implemented for SNP genomic relationships. The MNP approach is based on the result that the numerator matrix of each relationship matrix of Eqs. 14–16 can be expressed as a sum of the numerator matrices of all s small files, i.e.,

(28) $${\text{A}}_{\text{g}}={\text{W}}_{\alpha }\,{\text{W}}_{\alpha }^{\prime }/k_{\alpha }=\left(\sum_{i=1}^{s}{\text{W}}_{\alpha }^{i}\,{\text{W}}_{\alpha }^{i^{\prime }}\right)/k_{\alpha }$$

(29) $${\text{D}}_{\text{g}}={\text{W}}_{\delta }\,{\text{W}}_{\delta }^{\prime }/k_{\delta }=\left(\sum_{i=1}^{s}{\text{W}}_{\delta }^{i}\,{\text{W}}_{\delta }^{i^{\prime }}\right)/k_{\delta }$$

(30) $${\text{A}}_{\text{gh}}={\text{W}}_{\alpha \,h}\,{\text{W}}_{\alpha \,h}^{\prime }/k_{\alpha \,h}=\left(\sum_{i=1}^{s}{\text{W}}_{\alpha \,h}^{i}\,{\text{W}}_{\alpha \,h}^{i^{\prime }}\right)/k_{\alpha \,h}$$

where k_α_, k_δ_, and k_α*h*_ are defined by Eqs. 8–10. Different ${\text{W}}_{\alpha }^{\text{i}}\,{\text{W}}_{\alpha }^{i^{\prime }}$ matrices in Eq. 28, ${\text{W}}_{\delta }^{\text{i}}\,{\text{W}}_{\delta }^{\text{i}}$ in Eq. 29, and ${\text{W}}_{\alpha \,h}^{\text{i}}\,{\text{W}}_{\alpha \,h}^{\text{i}}$ in Eq. 30 are processed by different nodes and are saved separately as a binary file. These matrices are then added together to create each genomic relationship matrix using Eqs. 28–30. Each genomic relationship matrix for all haplotypes or SNPs is saved as a binary file. The GVCHAP analysis loads these saved matrices with negligible computing time and the main computing time required for the GVCHAP analysis becomes that for iterations. The MNP results in the same savings in computing time due to processing of the genomic relationship matrices as the two-step strategy (Table 3) and can be considered as a multi-node version of the two-step strategy. The unique advantage of the MNP approach is the removal of hardware limitation of a single node and the ability to use multiple nodes simultaneously, making what is undoable using a single node doable using multiple nodes. For the example of 35,745 individuals and 328,512 SNPs, a single node with 62 Gb memory could not complete the haplotype genomic relationship matrix, and this matrix could be created in about 3 h using MNP (Table 2).

### Heritability Tolerance to Reduce the Number of Iterations

With the removal of the computing bottleneck for processing haplotype genotypes by the two-step strategy and MNP method, the computing bottleneck becomes iterative solutions for GREML. The computing time per iteration increased from less than 1.5 min for 15,098 individuals to 22–29 min for 37,745 individuals (Table 2). The current version of GVCHAP uses shared memory parallel computing that can utilize all cores within a single node, but cannot use multiple nodes simultaneously. When AI-REML is used, GREML iterations generally converge fast, but EM-REML is used automatically when AI-REML fails and EM-REML may require many iterations to converge. To reduce the computing time required by EM-REML, GVCHAP implements two types of tolerance levels, heritability tolerance and variance component tolerance. Heritability tolerance can be substantially less stringent than variance component tolerance, e.g., 10^–6^ for heritability tolerance and 10^–8^ for variance component tolerance. Since heritability estimates are rarely reported with more than three decimal points, the 10^–6^ tolerance for heritability could significantly reduce the number of EM-REML iterations. With two types of tolerance levels, iteration stops when any of the two tolerance levels is reached.

### Challenge of Large Numbers of Individuals

The number of individuals is the limiting factor within each iteration because of matrix inversions and the need to store genomic relationship matrices for GREML. As the number of individuals increases, the sizes of the genomic relationship matrices and hence the required memory to store those matrices increase, and the computing time may increase rapidly. The choice of statistical model has a major impact on the memory requirement. Model 1 with haplotype additive effects and SNP additive and dominance effects requires the most memory, whereas Model 4 with haplotype additive effects requires the least memory among all models with haplotype effects. Therefore, Model 4 is the computationally competitive choice among Models 1–4 if Models 1–3 do not have substantially better prediction accuracy than Model 4. As shown in Table 2, increasing the number of individuals from 15,098 to 37,745 increased the computing time to 22–29 min from 82 s per iteration due to the larger genomic relationship matrices, 37,745 × 37,745 for 37,745 individuals versus 15,098 × 15,098 for 15,098 individuals. For this case, the matrix size increased 2.5 times and required 6.25 times as much memory to store each genomic relationship matrix. The ultimate computing solution to increase the GVCHAP capability for large numbers of individuals would be the use of distributed memory parallel computing, or parallel computing with massage passing interface (MPI) for using multiple nodes to reduce computing time and to use a large amount of memory. We have developed a MPI version of GVCBLUP (Wang et al., 2014a), and a MPI version of GVCHAP may be developed in the future.

## Conclusion

The GVCHAP program provides a capability for GBLUP and GREML to identify optimal prediction models using haplotypes based on structural and functional genomic information for genomic selection. The utility programs for data preparation and summary analysis of GVCHAP results eliminate most of the tedious and time-consuming data work. The entire pipeline provides an efficient and versatile computing tool to investigate candidate haplotype models utilizing structural and functional genomic information for genomic selection.

## Software Availability

The GVCHAP computing pipeline is available at http://animalgene.umn.edu.

## Data Availability Statement

All datasets generated for this study are included in the article/Supplementary Material.

## Author Contributions

YD conceived the study. DP was the author of the current version of GVCHAP and the utility programs. CW was the author of the initial version of GVCHAP. ZL was the author of the MNP program. CB and CT provided extensive evaluation that improved GVCHAP and the utility programs. ZL and DP evaluated computing time required by GVCHAP. DP, ZL, and YD prepared the user manual and prepared the manuscript.

## Conflict of Interest

The authors declare that the research was conducted in the absence of any commercial or financial relationships that could be construed as a potential conflict of interest.
