# Supplementary material for: GVCHAP: A Computing Pipeline for Genomic Prediction and Variance Component Estimation Using Haplotypes and SNP Markers
Source: Front Genet. 2020 Apr 7;11:282. doi: 10.3389/fgene.2020.00282 (PMC7154123; doi:10.3389/fgene.2020.00282)
Supplement: Supplementary file 1 [file Data_sheet_1.pdf]

***Supplementary Material*:**

GVCHAP: A Computing Pipeline for Genomic Prediction and Variance Component Estimation Using Haplotypes and SNP Markers

Dzianis Prakapenka[1], Chunkao Wang[1,2], Zuoxiang Liang[1] and Yang Da[1,*]

[1] Department of Animal Science, University of Minnesota, Saint Paul, MN 55108, USA; [2] Current address: Cobb-Vantress, Inc., Siloam Springs, AR 72761, USA.

GVCHAP USER MANUAL

VERSION 2.1

MARCH 13, 2020

1 Introduction

Haplotype analysis opens many opportunities to utilize structural and function information for genomic prediction and estimation, but requires large amounts of data preparation and summary analysis of results from a potentially large number of candidate models. The computing pipeline in this package provides capability for pre- and post-GVCHAP analysis, and reduces potentially tedious and time consuming haplotype analysis to a nearly automation process.

GVCHAP implements a multi-allelic haplotype mixed model that treats each haplotype block as a 'locus' and each haplotype within the haplotype block as an 'allele' (Da, 2015), and uses the GREML_CE program in the GVCBLUP package (Wang et al., 2014). The mixed model may include SNP additive and dominance effects and haplotype additive effects, with user flexibility to fit any or all of these three genomic effects. Haplotype dominance effects were coded but disabled in GVCHAP due to the large number of haplotype pairs that may exist in some haplotype blocks. The dominance effect of each haplotype pair requires all three genotypes to define, one heterozygous and two homozygous genotypes of the SNP, but one or both homozygous genotypes may be missing when the many haplotypes with small frequencies exist. For this reason, haplotype dominance effects are not allowed in GVCHAP. The computing pipeline consists of three components: data preparation, GVCHAP analysis, and summary analysis of GVCHAP results (Figure 1) (Prakapenka et al., 2020).

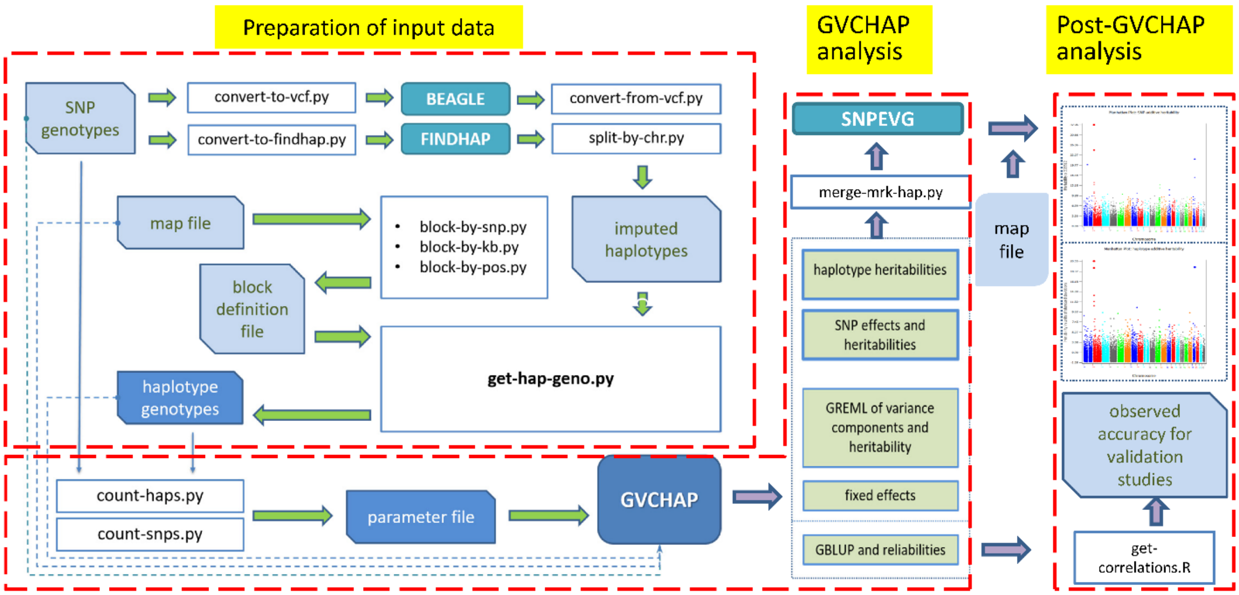

Figure 1. Structure of GVCHAP pipeline. The pipeline consists of three components, preparation of input data for haplotype analysis, GVCHAP analysis of genomic prediction and estimation, and post-GVCHAP analysis.

2 Data Preparation

The data preparation step starts with converting the SNP data into the input format for haplotype imputing using BEAGLE (Browning et al., 2018) or FINDHAP (VanRaden and Sun, 2014), then defining the haplotype blocks and genotypes, and configuring the parameter file that controls the execution of GVCHAP. To guide this process, a set of python scripts are provided. Each has a help file built in with the various options listed and described. To view the help file run the program with '-h' or '--help' flags.

2.1 Preparing input files for imputation

A set of utility scripts are provided to convert SNP data in GVCBLUP format to BEAGLE (Browning et al., 2018) or FINDHAP (VanRaden and Sun, 2014) input formats. BEAGLE uses VCF format, this step may be skipped if the data is already in VCF format. The haplotype output files of BEAGLE or FINDHAP are used to define haplotype genotypes.

> **convert-to-vcf.py**
> Purpose: converts SNP map and genotype files to VCF for imputing with BEAGLE
> Input: SNP genotype files in GVCHAP format
> Output: VCF format files

> **convert-to-findhap.py**
> Purpose: converts SN, map and genotype files to FINDHAP format for imputing with FINDHAP
> Input: SNP genotype files in GVCHAP format
> Output: FINDHAP input files

Box 1: Example of a haplotype file in vcf format used by BEAGLE

```
##fileformat=VCFv4.2
##fileDate=2019-11-13-09:27
##source=GVCHAP-converter
##FORMAT=<ID=GT,Number=1,Type=String,Description="Genotype">
#CHROM POS ID REF ALT QUAL FILTER INFO FORMAT Ind_1 Ind_2 Ind_3 Ind_4 Ind_5 Ind_6
Ind_7 Ind_8 Ind_9 Ind_10
1 485953  M_1  T C - - - GT 0|0 0|1 0|0 1|1 0|1 1|1 0|0 0|1 0|0 1|1
1 1666173 M_2  T C - - - GT 0|0 0|0 0|0 0|0 0|0 0|0 0|0 0|0 0|0 0|0
1 2753327 M_3  T C - - - GT 1|1 1|1 0|1 1|1 1|1 1|1 1|1 0|1 1|1 1|1
1 3078783 M_4  T C - - - GT 0|0 0|0 0|0 0|0 0|0 0|0 0|0 0|0 0|0 0|0
1 3186152 M_5  T C - - - GT 1|1 1|1 1|1 0|0 1|1 0|0 1|1 1|1 1|1 0|1
1 3186260 M_6  T C - - - GT 1|1 1|1 1|1 0|0 1|1 0|0 1|1 1|1 1|1 0|0
1 3270679 M_7  T C - - - GT 1|1 1|1 1|1 0|0 1|1 0|0 1|1 1|1 1|1 0|1
1 3830498 M_8  T C - - - GT 0|0 0|1 0|0 0|1 0|0 0|0 0|0 0|1 0|0 0|0
1 4609477 M_9  T C - - - GT 0|0 1|1 1|1 0|0 1|1 0|0 1|1 1|1 1|1 0|0
1 4764811 M_10 T C - - - GT 1|1 1|1 0|1 1|1 0|1 1|1 1|1 0|1 1|1 1|1
```

Box 1 is an example of a haplotype file imputed by BEAGLE in VCF file format. The structure of the file is one marker position per row with individual samples and their alleles starting in the 10th column.

FINDHAP input files require genotypes.txt and chromosome.data files which can be created using **convert-to-findhap.py** script. A pedigree.file is also required and must be supplied by the user.

Box 2: Example of a haplotypes.txt file from FINDHAP

```
Ind_1        1      10      1 1 1 1 2 2 1 1 2 2 2 2 2 2 1 1 1 1 2 2
Ind_2        1      10      1 2 1 1 2 2 1 1 2 2 2 2 2 2 1 2 2 2 2 2
Ind_3        1      10      1 1 1 1 1 2 1 1 2 2 2 2 2 2 1 1 2 2 1 2
Ind_4        1      10      2 2 1 1 2 2 1 1 1 1 1 1 1 1 1 2 1 1 2 2
Ind_5        1      10      1 2 1 1 2 2 1 1 2 2 2 2 2 2 1 1 2 2 1 2
Ind_6        1      10      2 2 1 1 2 2 1 1 1 1 1 1 1 1 1 1 1 1 2 2
Ind_7        1      10      1 1 1 1 2 2 1 1 2 2 2 2 2 2 1 1 2 2 2 2
Ind_8        1      10      1 2 1 1 1 2 1 1 2 2 2 2 2 2 1 2 2 2 1 2
Ind_9        1      10      1 1 1 1 2 2 1 1 2 2 2 2 2 2 1 1 2 2 2 2
Ind_10       1      10      2 2 1 1 2 2 1 1 1 2 1 1 1 2 1 1 1 1 2 2
```

Box 2 is an example of haplotype.txt file imputed from FINDHAP. The haplotype.txt file is structured as one individual per row with the paternal and maternal alleles for each SNP.

2.2 Converting imputed data to input files for defining haplotype genotypes

The following set of utility scripts are provided to convert the output of BEAGLE and FINDHAP for another utility program to define haplotype genotype files.

> **convert-from-vcf.py**
> Purpose: converts haplotype files in VCF format to the format for get-hap-geno.py
> Input: imputed vcf chromosome files
> Output: haplotype files in GVCHAP format split by chromosome (**Box 3**)

> **split-by-chr.py**
> Purpose: converts and splits by chromosome the output of FINDHAP imputing program for get-hap-geno.py
> Input: findhap.f90 output files (haplotypes.txt or (hap.list and hap.found), chromosome.data file, genotypes.filled file)
> Output: haplotype files in GVCHAP format split by chromosome (**Box 3**)

Box 3: Example of output file from convert-from-vcf.py or split-by-chr.py

```
Ind_1  1 1 1 1 2 2 1 1 2 2 2 2 2 2 1 1 1 1 2 2
Ind_2  1 2 1 1 2 2 1 1 2 2 2 2 2 2 1 2 2 2 2 2
Ind_3  1 1 1 1 1 2 1 1 2 2 2 2 2 2 1 1 2 2 1 2
Ind_4  2 2 1 1 2 2 1 1 1 1 1 1 1 1 1 2 1 1 2 2
Ind_5  1 2 1 1 2 2 1 1 2 2 2 2 2 2 1 1 2 2 1 2
Ind_6  2 2 1 1 2 2 1 1 1 1 1 1 1 1 1 1 1 1 2 2
Ind_7  1 1 1 1 2 2 1 1 2 2 2 2 2 2 1 1 2 2 2 2
Ind_8  1 2 1 1 1 2 1 1 2 2 2 2 2 2 1 2 2 2 1 2
Ind_9  1 1 1 1 2 2 1 1 2 2 2 2 2 2 1 1 2 2 2 2
Ind_10 2 2 1 1 2 2 1 1 1 2 1 1 1 2 1 1 1 1 2 2
```

2.3 Defining haplotype blocks

Haplotype genotypes are required for GVCHAP analysis. The first step to define haplotype genotypes is to define haplotype blocks, where each block is treated as a 'locus' and each haplotype within the block as an 'allele'. Functional and structural genomics information can be used to define haplotype blocks.

The following Python programs implement three methods for defining haplotype blocks.

block-by-kb.py:
Purpose: Generate haplotype block information files by fixed distance in kb
Input: map file, size of block
Output: hap_block and hap_block info files

block-by-snp.py:
Purpose: Generate haplotype block information files by fixed number of SNPs
Input: map file, number of SNPs per block
Output: hap_block and hap_block info files

block-by-pos.py:
Purpose: Generate haplotype block information files with a list of begin and end positions to provide flexibility to use functional and structural information to define haplotype blocks
Input: list of blocks in the format "chr:beginPos-endPos", map file
Output: hap_block and hap_block info files

The hap_block and hap_block_info files generated by these programs are editable or can be created by the user for custom definitions of haplotype blocks. An example of a hap_block file for a chromosome with 4 SNP blocks:

Box 4: Example of haplotype blocks

```
blk1   0 3
blk2   4 7
blk3   8 9
```

2.4 Producing haplotype genotype files from defined blocks

GVCHAP provides a utility program, **get-hap-geno.py**, to generate the haplotype files from user provided input files of imputed SNP genotypes and input files for the positions of haplotype blocks of each chromosome.

get-hap-geno.py:
Purpose: generate haplotype genotype files using block definitions
Input: hap_info_files, haplotype files, (coding for missing alleles)
Output: hap_geno files

The haplotypes files are provided by the user and are specified in the parameter file. Each file is for one chromosome, and the number of haplotype files must be the same as the number of SNP files. Each haplotype block takes two columns in the haplotype file, where each haplotype is treated as an 'allele' and each haplotype block is treated as a 'multi-allelic locus' by GVCHAP (**Box 5**).

Box 5: Example of a haplotype genotype input file for GVCHAP

```
ID hap_1_1 hap_1_1 hap_1_2 hap_1_2 hap_1_3 hap_1_3
Ind_1 1 1 1 1 1 1
Ind_2 1 2 1 2 2 2
Ind_3 3 1 1 1 3 2
Ind_4 2 2 3 4 1 1
Ind_5 1 2 1 1 3 2
Ind_6 2 2 3 3 1 1
Ind_7 1 1 1 1 2 2
Ind_8 3 2 1 2 3 2
Ind_9 1 1 1 1 2 2
Ind_10 2 2 3 5 1 1
```

The general format of the haplotype files represented by **Box 5** is as follows:

[Sample ID] [hap_1_1] [hap_1_1] [hap_1_2] [hap_1_2] [hap_1_3] [hap_1_3]…
[Sample ID] [locus_allele_1] [locus_allele_1] [locus_allele_2] [locus_allele_2]

2.5 Configuring parameter file

The parameter file is configured manually based on a template (Explained in Section 3). For the more tedious tasks such as listing SNP and haplotype genotype files with the counts of markers/blocks the following utilities are provided.

> **count-haps.py**
> Purpose: generate a list of haplotype chromosomes with number of haplotype blocks for each
> Input: path to haplotype genotype files, (flag to optionally sort by any number in the file name)
> Output: lists chromosome files with appropriate parameter prefix and haplotype block count

> **count-snps.py**
> Purpose: generate a list of SNP chromosomes with number of SNPs for each
> Input: path to SNP genotype files, (flag to optionally sort by any number in the file name)
> Output: lists chromosome files with appropriate parameter prefix and SNP count

3 GVCHAP Input Files

GVCHAP requires the following files to run:

- Parameter file
- Phenotype file
- SNP genotype files
- Haplotype genotype files
- SNP map file

3.1 Parameter file (user defined file name)

A parameter file with a user defined name ("*gparameter.dat*" is the default name if nothing is provided as argument) is required to run GVCHAP. The parameter file specifies a mixed model, the input file names for the SNP, haplotype and phenotype input data; the output file names; how to use the combination of REML and AI-REML; the maximum number of iterations allowed; and code for missing phenotypic observations of selection candidates or validation population. Box 1 explains each line in the parameter file.

The format of the parameter file is one '**keyword**' per line. Anything after a pound sign (#) is not read by the program and can be used for comments. The **keywords** in **Box 1** are bolded.

The following explains each keyword.

num_iter n
A positive integer n represents the maximum number of iterations the user allows GVCHAP for calculating GREML. If '0' is used as the iteration number, GREML is skipped and GBLUP is calculated using user provided starting values for variance components.

tolerance_var n
A positive double n represents the tolerance level to declare convergence of GREML iteration. Iteration stops when the difference between variance components from iteration j and iteration j+1 becomes less than or equal to n, which often can be 10^{-8}.

tolerance_heritability n
A positive double n represents the tolerance level to declare convergence of GREML iteration. Iteration stops when the difference between heritability estimates from iteration j and iteration j+1 becomes less than or equal to n, which often can be 10^{-6}.

missing_phen_val n
Integer n represents the code for missing phenotypic observations. Choose a value different from any true phenotypic observations.

missing_hap_val n
Integer n represents the code for missing haplotype blocks. Choose a value different from any true haplotype blocks.

use_ai_reml n
A positive integer n represents the number of EM-REML iterations before starting AI-REML.

num_ind n
A positive integer n represents the number of individuals in the phenotype and genotype files.

traits_col n
A positive integer n represents the column of phenotypic observations in the pheonotype file.

factors_pos n1 n2 …
A set of positive integers (n1,n2,…) represents the columns of fixed non-genetic factors in the pheonotype file. These are treated as classification variables in the statistical model (such as herd, year, season and sex). The number of levels of a fixed non-genetic factor should be substantially smaller than the number of phenotypic observations. A variable with many levels should be declared as a continuous variable (see covar_pos). Declaring a variable with many levels as a classification variable could result in an over-fitted model and cause GVCHAP to fail.

covar_pos n1 n2 …
A set of positive integers (n1,n2,…) represents the columns of covariables. Covariables are non-genetic factors but are treated as continuous variables in the statistical model (such as age and body weight). As a general rule, a variable with many levels should be declared as a ‘covariable’. Declaring a variable with many levels as a classification variable could result in an over-fitted model and cause GVCHAP to fail.

phenotype str
A string str represents the full path to the phenotype file and the name of the phenotype file.

var_snp_a n
A positive number n represents the starting value of the SNP additive variance component. A ‘0’ starting value can be used to skip GREML and GBLUP for this effect.
var_snp_d n
A positive integer n represents the starting value of the SNP dominance variance component. A ‘0’ starting value can be used to skip GREML and GBLUP for this effect.
var_snp_e n (required)
A positive integer n represents the starting value of the residual variance component. This value is required and cannot be skipped.

var_hap_a n
A positive integer n represents the starting value of the haplotype additive variance component. A ‘0’ starting value can be used to skip GREML and GBLUP for this effect.

var_hap_d n
A positive integer n represents the starting value of the haplotype dominance variance component. A ‘0’ starting value can be used to skip GREML and GBLUP for this effect.

Setting all starting values of the SNP variance components to ‘0’ or not including the keyword skips all SNP effects. Haplotype effects can be skipped in a similar manner. Variances with positive starting values are estimated iteratively using GREML. GBLUP of additive and dominance effects as well as the total genotypic value are calculated using the estimates from the last of GREML iterations.

geno_snp *n str*
A string *str* represents the full path to a SNP genotype file for a single chromosome. A positive integer *n* represents the number of SNPs in the chromosome file. This keyword should be repeated for each chromosome.

geno_hap *n str*
A string *str* represents the full path to a haplotype genotype file for a single chromosome. A positive integer *n* represents the number of blocks in the chromosome file. This keyword should be repeated for each chromosome.

The number of chromosomes must be consistent across haplotype and SNP analysis (i.e. the number of SNP genotype files and haplotype genotype files must be the same if doing combined SNP+haplotype analysis).

map_file *str*
A string *str* represents the full path to the map file and the name of the map file. The map file must have three columns titled 'SNPID Chr Position'.

out_greml *str* (required)
A string *str* represents the full path to the GREML output file for SNPs and haplotypes.

out_gblup *str* (required)
A string *str* represents the full path to the GBLUP output file for SNPs and haplotypes.

output_fixed_effect *str*
A string *str* represents the full path to the output file for estimated fixed non-genetic effects.

output_hap_effect *str*
A string *str* represents the full path to the output file for haplotype additive and dominance heritabilities.

output_mrk_effect *str*
A string *str* represents the full path to the output file for SNP additive and dominance heritabilities.

out_rel *str*
A string *str* represents the full path to the output file for genomic relationships.

Box 6: Example of a parameter file for GVCHAP

```
# comments are designated with the pound sign '#', nothing on a line
# after the pound sign is read by the program
# SNP and haplotype files will be read in the order given
# the order of other keywords does not matter

num_iter 1000                   # number of iterations for GREML,
                                # set to 0 to skip GREML

tolerance_var 1.0E-08           # variance tolerance threshold
tolerance_heritability 1.0E-06  # heritability tolerance threshold

# starting values of SNP variance components
# set var_snp_a or var_snp_d to 0 to skip
var_snp_a 1     # starting value for SNP additive variance
var_snp_d 2     # starting value for SNP dominance variance
var_snp_e 3     # starting value for residual variance component
                # to disable additive or dominance analysis, remove
                # the corresponding line or add '#' to front of it
                # to comment it out

#starting value of haplotype additive variance component
var_hap_a 50    # starting values of haplotype VA
                # to disable haplotype analysis, remove this line
                # or add '#' to front of this line

phenotype sim_traits.txt  # name of input phenotype file

missing_phen_val 9999     # '9999' is user defined code for missing
                          # observation in phenotype data
missing_hap_val  -9999    # '-9999' is user defined code for missing
                          # observation in haplotype data

use_ai_reml 3             # '3' to use AI-REML starting with
                          # iteration 3, '0' to use EM-REML

num_ind 4364     # number of individuals

traits_col 6     # column number for the trait in the phenotype file

# covariables and non-genetic factors
factors_pos 2 3    # column numbers of non-genetic fixed factors
                   # in phenotype file
covar_pos 4 5      # column numbers of covariables in phenotype file

# SNP genotype files
# keyword number_of_SNPs path_to_file
geno_snp 33905 chr01.dat
geno_snp 35537 chr02.dat

# Haplotype genotype files
# keyword number_of_hap_blocks path_to_file
geno_hap 1211 hap_geno_chr1.dat
geno_hap 1117 hap_geno_chr2.dat

map_file map.txt        # map file with three columns
```

```
                          # titled 'SNPID Chr Position'

# required output files
out_greml greml.out     # user defined file name of the GREML data
out_gblup gblup.out     # user defined file name of the GBLUP data

# optional output files
output_fixed_effect fixed_effect.out    # file name for covariable
                                        # and non-genetic effects
output_hap_heritabilities hap_herit.snpe     # file name for haplotype
                                             # heritabilities
output_mrk_effect mrk_effect.snpe       # file name for SNP
                                        # heritabilities
out_rel  rel.txt       # output genomic relationships to rel.txt

save_g run1            # save genomic relationship matrix as 'run1'
#load_g run1           # load genomic relationship matrix from 'run1'
```

3.2 Chromosome files of SNP markers

The chromosome files are provided by the user and are specified in the parameter file. Each file is for one chromosome. Alternatively, the SNP genotypes can be placed in one file, and output is the same if SNPs are in the same order. One SNP takes one column in the SNP file. The coding for SNP genotypes is always 0 = A1A1, 1 = A1A2, and 2 = A2A2, where '0' and '2' denote the two homozygous genotypes and '1' denotes the heterozygous genotype. Any other number denotes a missing SNP genotype and results in zero in matrix construction.

The general format of the SNP input files is as follows:

[name of each column]
[Sample ID], [locus1], [locus2], [locus3], [locus4],…

Box 7 is an example of a SNP input file for GREML.

Box 7: Example of a SNP genotype file

```
ID M_1 M_2 M_3 M_4 M_5 M_6 M_7 M_8 M_9 M_10
Ind_1 0 0 2 0 2 2 2 0 0 2
Ind_2 1 0 2 0 2 2 2 1 2 2
Ind_3 0 0 1 0 2 2 2 0 2 1
Ind_4 2 0 2 0 0 0 0 1 0 2
Ind_5 1 0 2 0 2 2 2 0 2 1
Ind_6 2 0 2 0 0 0 0 0 0 2
Ind_7 0 0 2 0 2 2 2 0 2 2
Ind_8 1 0 1 0 2 2 2 1 2 1
Ind_9 0 0 2 0 2 2 2 0 2 2
Ind_10 2 0 2 0 1 0 1 0 0 2
```

3.3 Chromosome files of haplotype genotypes

The chromosome files of haplotype genotypes contains the individual ID and haplotype genotypes per row, below the heading of the file. Each haplotype block takes two columns. The general format of the SNP input files is as follows:

[name of each column]
[Sample ID], [locus1], [locus2], [locus3], [locus4],…

Box 8: Example of a haplotype genotype file

```
ID hap_2_1 hap_2_1 hap_2_2 hap_2_2 hap_2_3 hap_2_3
Ind_1 1 1 1 1 1 2
Ind_2 2 3 2 2 1 1
Ind_3 1 1 1 1 1 1
Ind_4 1 4 1 3 3 1
Ind_5 2 5 2 4 1 2
Ind_6 2 2 2 2 1 2
Ind_7 1 4 1 1 3 1
Ind_8 1 1 1 3 1 1
Ind_9 1 1 1 1 1 1
Ind_10 2 3 2 2 3 1
```

3.4 Phenotype file

The phenotype file contains quantitative traits, fixed non-genetic effects such as gender, herd, year, season, blocks, treatment and living conditions, and covariables such as body weight and age. A trait can be used as a covariable. For example, birth weight can be a trait can be a covariable for analyzing the weight at six month old. The name of the phenotype file is determined by the user and is specified in the parameter file. The general format of the phenotype file for GREML is as follows:

[name of each column]
[Sample ID] [fixed effects, one fixed factor per column] [covariables, one covariable per column] [trait 1] [trait 2] [trait 3] …

Box 9: Example of a phenotype input file

```
sample_id      factor1  factor2   cov1    cov2    trait1   trait1_val
Ind_1          1        B         1.1     4       1.7      1.7
Ind_2          1        A         2.3     3       1.2      -9999
Ind_3          2        B         0.7     8       1.3      1.3
Ind_4          2        B         0.3     2       2.1      2.1
Ind_5          1        A         3.5     5       2.3      -9999
Ind_6          2        A         1.5     9       0.3      0.3
Ind_8          3        B         2.8     7       1.5      1.5
Ind_9          3        A         3.6     4       3.8      -9999
Ind_10         2        A         5.1     8       3.0      3.0
```

WARNINGS: The sample IDs must match those in each chromosome file.

3.5 SNP map file

The SNP map file contains SNP name, chromosome number and physical SNP position. This file is used for the ‘mrk_effect.snpe’ file that can be used to generate Manhattan plots and chromosome graphs of SNP heritability and effects.

Box 10: Example of SNP map file

SNPID	Chr	Position
M_1	1	485953
M_2	1	1666173
M_3	1	2753327
M_4	1	3078783
M_5	1	3186152
M_6	1	3186260
M_7	1	3270679
M_8	1	3830498
M_9	1	4609477
M_10	1	4764811
M_11	2	730589
M_12	2	755444
M_13	2	841463
M_14	2	841675
M_15	2	851161
M_16	2	1281122
M_17	2	1360754
M_18	2	1723802

4 Output Files of GVCHAP

GVCHAP produces six output files with the option to produce any or all six output files with user-specified file names. **The GBLUP file** contains GBLUP of SNP and haplotype additive, dominance and total genetic values with associated reliability for all individuals with and without phenotypic observations. **The GREML file** contains estimates of variance components and heritabilities for SNP and haplotype additive and dominance values. **The marker effect file** contains GBLUP of SNP additive and dominance effects, and additive, dominance and total heritabilities for each SNP along with the chromosome map positions for graphing using SNPEVG2. **The haplotype effect file** contains GBLUP of haplotype additive and dominance effects, and additive, dominance and total heritabilities for each haplotype block for graphing using SNPEVG2 after an update for map positions. **The fixed effect file** contains best linear unbiased estimates (BLUE) of fixed effects of non-genetic effects or fixed genetic effects that could be combined with GBLUP for genetic selection. The log is printed to standard out (in the terminal) and can be captured with a redirect (">") to a file. It records most steps of the GVCHAP calculations and computing time for each task.

4.1 GREML output file (greml.out)

The GREML procedure in GVCHAP estimates additive and dominance variances of SNPs and haplotypes, residual variance, additive and dominance heritabilities, and heritability in the broad sense of SNPs and haplotypes (**Table 6**).

Box 11: Example of heritability estimates with standard error (SE) in the GREML output file of GVCHAP

```
h2_A: Additive heritability, SE:                1.50e-001, 3.20e-001
h2_D: Dominance heritability, SE:               3.38e-001, 4.23e-001
h2_AH: Haplotype additive heritability, SE:     3.69e-001, 6.16e-001
H2: Heritability in the broad sense, SE:        9.88e-001, 8.38e-001
```

In addition to the heritability estimates, the GREML output file also summarizes information of SNP and phenotypic data including the number of SNPs, number of chromosomes, number of individuals with phenotypic observations, number of individuals without observations, total running time, and estimates of variance components and observed tolerance levels at each iteration.

4.2 GBLUP output file (gblup.out)

GBLUP and reliability of breeding values, dominance deviations and genetic values of SNPs and haplotypes for all individuals including individuals with phenotypic observation ('T' in Train./Valid. column) and individuals without phenotypic observations ('V' in Train./Valid. Column) (**Box 12**).

Box 13: Example of output file for GBLUP: SNP_a + Hap_a

```
ID  GBLUP_A       Reliability_A   GBLUP_AH      Reliability_AH   GBLUP_G       Reliability_G Train./Valid.
9    3.458775e-04  8.287500e-03  -2.597149e-01  6.249874e-01  -2.593690e-01  6.328995e-01   T
12 -5.176884e-03  8.902630e-03  -8.999710e-02  6.604898e-01  -9.517399e-02  6.689748e-01   T
24 -1.504806e-02  8.409952e-03  -1.121016e+00  6.264837e-01  -1.136065e+00  6.344945e-01   T
35  1.748771e-02  8.936526e-03   1.944280e+00  6.643663e-01   1.961767e+00  6.728923e-01   T
36  3.783208e-03  8.476587e-03   1.755655e-01  6.292247e-01   1.793487e-01  6.372796e-01   T
37 -7.317768e-03  8.441457e-03  -7.577906e-01  6.198264e-01  -7.651084e-01  6.278601e-01   T
51 -8.636336e-04  3.946032e-03   1.857288e-02  2.561144e-01   1.770924e-02  2.596664e-01   V
```

60	2.191553e-02	8.765625e-03	1.567232e+00	6.528188e-01	1.589147e+00	6.611995e-01	T
63	-1.858041e-02	8.947421e-03	-1.287023e+00	6.629195e-01	-1.305604e+00	6.714503e-01	T
64	1.252362e-02	1.384385e-03	6.019094e-01	6.529024e-02	6.144331e-01	6.629012e-02	V
65	-1.236164e-02	8.329126e-03	-1.096748e+00	6.215390e-01	-1.109110e+00	6.294556e-01	T
66	9.867920e-03	8.314020e-03	9.328004e-01	6.182038e-01	9.426684e-01	6.261111e-01	T
74	-2.924892e-03	8.370108e-03	-5.420744e-01	6.245559e-01	-5.449993e-01	6.325234e-01	T

Box 14: Example of output file for GBLUP: SNP_d + Hap_a

ID	GBLUP_D	Reliability_D	GBLUP_AH	Reliability_AH	GBLUP_G	Reliability_G	Train./Valid.
9	7.424680e-03	3.371856e-02	-2.653895e-01	6.167149e-01	-2.579648e-01	6.475626e-01	T
12	2.822698e-02	3.410643e-02	-9.987360e-02	6.517298e-01	-7.164662e-02	6.798464e-01	T
24	-7.618994e-02	3.437513e-02	-1.094087e+00	6.189690e-01	-1.170277e+00	6.482572e-01	T
35	3.912322e-02	3.503329e-02	1.935242e+00	6.563723e-01	1.974365e+00	6.830886e-01	T
36	1.155638e-02	3.422684e-02	1.742024e-01	6.206441e-01	1.857588e-01	6.519114e-01	T
37	-5.242978e-02	3.390447e-02	-7.413362e-01	6.117215e-01	-7.937660e-01	6.426115e-01	T
51	-6.313029e-03	5.398769e-03	1.708513e-02	2.533727e-01	1.077210e-02	2.520267e-01	V
60	5.631798e-02	3.369428e-02	1.542891e+00	6.442011e-01	1.599209e+00	6.727670e-01	T
63	1.283644e-02	3.510237e-02	-1.292005e+00	6.552641e-01	-1.279168e+00	6.814615e-01	T
64	-1.047166e-02	2.174208e-03	6.008660e-01	6.452312e-02	5.903943e-01	6.154856e-02	V
65	-3.789740e-02	3.379974e-02	-1.090274e+00	6.133986e-01	-1.128171e+00	6.441977e-01	T
66	2.934607e-02	3.355423e-02	9.280413e-01	6.103121e-01	9.573874e-01	6.407897e-01	T
74	1.510771e-03	3.414411e-02	-5.478755e-01	6.162381e-01	-5.463647e-01	6.472304e-01	T

4.3 Haplotype heritabilities (hap_herit.snpe)

Currently the output file of haplotype heritabilities (**Box 14**) is an intermediate step for producing Manhattan plots and chromosome graphs after merging with the file of SNP effects and heritabilities using ‘merge-mrk-hap.py’ utility program.

Box 14: Example of output file for haplotype heritabilities

HAPID	h2_hap_AH	h2_hap_std_AH	h2_hap_AH_nh
0	2.116055E-05	-7.271836E-02	5.795240E-01
1	3.541547E-05	1.128774E+00	9.699235E-01
2	2.672610E-05	3.963802E-01	7.319477E-01
3	3.558114E-05	1.142738E+00	9.744607E-01
4	3.128970E-05	7.810291E-01	8.569310E-01
5	2.747003E-05	4.590835E-01	7.523217E-01
6	1.576249E-05	-5.277001E-01	4.316873E-01
7	2.612204E-05	3.454669E-01	7.154043E-01
8	3.787041E-05	1.335691E+00	1.037157E+00
9	4.785511E-05	2.177263E+00	1.310608E+00
10	1.322768E-05	-7.413491E-01	3.622665E-01

4.4 SNP effects and heritabilities (mrk_effect.snpe)

SNP effects estimated as the BLUP of SNPs along with SNP heritabilities calculated at the end of the GREML iterations are printed in the output file of SNP effects and heritabilities, named ‘mrk_effect.snpe’ in the example of the parameter file. SNP additive and dominance effects are printed as the original SNP BLUP values and the absolute SNP BLUP values, and SNP heritabilities are printed as the original heritability values and standardized heritability values. The purpose of this file is to produce Manhattan plots and chromosome graphs using SNPEVG2, as to be described later.

Box 15: Example of output file for SNP effects and heritabilities: SNP_a

SNPID	Chr	Position	Effect_A	m_effect_A	Effect_A2	m_effect_A2	h2_mrk_A	m_h2_mrk_A	H2_mrk
rs3094315	1	817186	2.864238e-03	9.409364e+02	2.864238e-03	9.409364e+02	9.437527e-08	3.100341e-02	9.437527e-08
rs4040617	1	843942	2.102765e-03	6.907834e+02	2.102765e-03	6.907834e+02	5.086528e-08	1.670986e-02	5.086528e-08
rs2980300	1	850609	1.892590e-03	6.217384e+02	1.892590e-03	6.217384e+02	4.120529e-08	1.353643e-02	4.120529e-08
rs4075116	1	1068249	-7.593908e-04	-2.494690e+02	7.593908e-04	2.494690e+02	6.633929e-09	2.179325e-03	6.633929e-09
rs6685064	1	1275912	-5.656291e-06	-1.858159e+00	5.656291e-06	1.858159e+00	3.680473e-13	1.209080e-07	3.680473e-13
rs3766180	1	1542773	-1.889700e-03	-6.207890e+02	1.889700e-03	6.207890e+02	4.107955e-08	1.349513e-02	4.107955e-08
rs6603791	1	1565561	-2.033705e-04	-6.680964e+01	2.033705e-04	6.680964e+01	4.757906e-10	1.563029e-04	4.757906e-10
rs7540231	1	1570655	-1.997268e-05	-6.561265e+00	1.997268e-05	6.561265e+00	4.588945e-12	1.507524e-06	4.588945e-12

Box 16: Example of output file for SNP effects and heritabilities: SNP_a + SNP_d

SNPID	Chr	Position	Effect_D	m_effect_D	Effect_D2	m_effect_D2	h2_mrk_D	m_h2_mrk_D	H2_mrk
rs3094315	1	817186	6.254768e-04	2.054766e+02	6.254768e-04	2.054766e+02	1.025220e-09	3.367971e-04	1.025220e-09
rs4040617	1	843942	4.267979e-03	1.402082e+03	4.267979e-03	1.402082e+03	4.773526e-08	1.568160e-02	4.773526e-08
rs2980300	1	850609	5.360683e-03	1.761049e+03	5.360683e-03	1.761049e+03	7.530693e-08	2.473923e-02	7.530693e-08
rs4075116	1	1068249	1.871331e-03	6.147548e+02	1.871331e-03	6.147548e+02	9.176901e-09	3.014722e-03	9.176901e-09
rs6685064	1	1275912	-2.931672e-03	-9.630894e+02	2.931672e-03	9.630894e+02	2.252296e-08	7.399062e-03	2.252296e-08
rs3766180	1	1542773	9.005749e-04	2.958497e+02	9.005749e-04	2.958497e+02	2.125369e-09	6.982092e-04	2.125369e-09
rs6603791	1	1565561	7.983894e-03	2.622805e+03	7.983894e-03	2.622805e+03	1.670414e-07	5.487511e-02	1.670414e-07
rs7540231	1	1570655	-6.909883e-04	-2.269979e+02	6.909883e-04	2.269979e+02	1.251227e-09	4.110430e-04	1.251227e-09

4.5 Fixed non-genetic effects (fixed_effect.out)

This output file contains estimated fixed non-genetic effects by the best leaner unbiased estimation (BLUE) at the end of the GREML iterations (**Box 17**).

Box 17: Example of estimates of fixed non-genetic effects

Fixed effect	Level name	Level	Value
0	mu	1	1.165615e+01
1	1	0	6.086162e+00
1	2	1	5.569993e+00
2	0	0	-4.608519e-01
2	1	1	8.125373e-01
3	1	0	-2.209107e-05
3	0	1	-8.386171e-02
3	2	2	-8.994692e-02
4	Covariable	1	2.137628e-02
5	Covariable	1	2.550369e-03
6	Covariable	1	7.875365e-02
7	Covariable	1	1.001592e-02
8	Covariable	1	-7.397074e-03

ID	XB	Train./Valid.
9	1.401006e+02	T
12	1.898920e+02	T
24	1.624787e+02	T
35	1.717123e+02	T
36	1.873999e+02	T
37	1.443500e+02	T
51	0.000000e+00	V

4.6 The log output

The log of each run is printed to stdout (**Box 18**). If desired, the log can be saved using bash redirection:

```
./gvchap parameter.txt > run1.log
```

Box 18: Example of log output (run1.log)

```
GVCHAP Version 4.0.0
The local date and time is: Tue Feb 25 13:52:20 2020

reading parameter file run_save.txt ... done.

==========parameters list============

Number of iterations:                                                     100
Initial variances:
  SNP Additive:                                                             1
  SNP Dominance:                                                            2
  Haplotype Additive:                                                      15
  Residual:                                                                 3
Variance component tolerance:                                           1e-08
Heritability tolerance:                                                 1e-06
Phenotype file:                                                 phenotype.txt
Position of trait column:                                                   6
No. cross factors:                                                          2
Position of cross factors:                                                  2 3
No. covariables:                                                            1
Position of covariables:                                                    4
No. genotyped individuals:                                                100
No. chromosomes:                                                            2
  No. mrk and name:                                         5350 snp_geno.dat
  No. mrk and name:                                         5350 snp_geno.dat
No. haplotype files:                                                        2
  No. hap and name:                                                9 hap_geno
  No. hap and name:                                               9 hap_geno1
Definition of Q:                                                            2
missing_phen_val:                                                        9999
missing_hap_val:                                                        -9999
use_ai_reml:                                                                1
iter_ai_reml_start:                                                         3
variance output file:                                   ./out_save/greml.out
GEBV output file:                                       ./out_save/gblup.out
fixed effects file:                              ./out_save/fixed_effect.txt
marker effects file:                               ./out_save/mrk_effect.txt
haplotype heritabilities file:              ./out_save/hap_heritabilities.txt
map file:                                                             map.txt
save_g:                                                                  test
=========end of parameters============

========finished para read============

========finished defQ read============
reading genotype file snp_geno.dat ...
processing SNP file took: 0 seconds.
```

```
reading genotype file snp_geno.dat ...
processing SNP file took: 0 seconds.

saving SNP data to test
       saving indgeno to test.indgeno
       saving g matrix to test.g.A
       saving g diag to test.g.A
       saving g matrix to test.g.D
       saving g diag to test.g.D

all SNP file processing took:  1 seconds.

total number of markers is: 10700

========finished geno read============
reading haplotype file hap_geno ...
processing haplotype file took: 0 seconds.

reading haplotype file hap_geno1 ...
processing haplotype file took: 0 seconds.

saving haplotype data to test
       saving indgeno to test.indgeno
       saving haplotype matrix to test.g.AH

all haplotype file processing took: 0 seconds.


========finished hap read=============
Number of phenotypic observations is 95
categorical variable 1 has 3 levels.
categorical variable 2 has 2 levels.

========finished phen read============


======== GREML begins ======

Iteration: 1
EM-REML
VA     = 5.941522e+02   Tolerance_VA     = 5.931522e+02
VD     = 1.214852e+03   Tolerance_VD     = 1.212852e+03
VAH    = 8.742678e+03   Tolerance_VAH    = 8.727678e+03
VE     = 1.767160e+03   Tolerance_VE     = 1.764160e+03
Time for this iteration is : 0 seconds

Iteration: 2
EM-REML
VA     = 6.015310e+02   Tolerance_VA     = 7.378772e+00
VD     = 1.256667e+03   Tolerance_VD     = 4.181496e+01
VAH    = 8.683593e+03   Tolerance_VAH    = 5.908507e+01
VE     = 1.773567e+03   Tolerance_VE     = 6.406553e+00

h2_A   = 4.884397e-02   Tolerance_h2_A   = 6.127997e-04
h2_D   = 1.020407e-01   Tolerance_h2_D   = 3.423255e-03
h2_AH  = 7.051027e-01   Tolerance_h2_AH  = 4.596855e-03
```

```
H2     = 8.559874e-01   Tolerance_H2   = 5.607999e-04
Time for this iteration is : 0 seconds

Iteration: 3
AI-REML
VA     = 1.630424e+03   Tolerance_VA     = 1.028893e+03
VD     = 4.135278e+03   Tolerance_VD     = 2.878611e+03
VAH    = 5.920270e+03   Tolerance_VAH    = 2.763323e+03
VE     = 4.657917e+02   Tolerance_VE     = 1.307775e+03

h2_A   = 1.341718e-01   Tolerance_h2_A   = 8.532785e-02
h2_D   = 3.403027e-01   Tolerance_h2_D   = 2.382620e-01
h2_AH  = 4.871943e-01   Tolerance_h2_AH  = 2.179085e-01
H2     = 9.616688e-01   Tolerance_H2   = 1.056814e-01
Time for this iteration is : 0 seconds

Iteration: 4
EM-REML
VA     = 1.655933e+03   Tolerance_VA     = 2.550885e+01
VD     = 4.172655e+03   Tolerance_VD     = 3.737642e+01
VAH    = 5.976424e+03   Tolerance_VAH    = 5.615375e+01
VE     = 4.695938e+02   Tolerance_VE     = 3.802094e+00

h2_A   = 1.349072e-01   Tolerance_h2_A   = 7.354234e-04
h2_D   = 3.399421e-01   Tolerance_h2_D   = 3.606423e-04
h2_AH  = 4.868934e-01   Tolerance_h2_AH  = 3.009251e-04
H2     = 9.617427e-01   Tolerance_H2   = 7.385598e-05
Time for this iteration is : 0 seconds

.
.
.

Iteration: 99
EM-REML
VA     = 1.950104e+03   Tolerance_VA     = 7.852893e-01
VD     = 4.175608e+03   Tolerance_VD     = 5.837571e-01
VAH    = 5.765877e+03   Tolerance_VAH    = 5.118697e-01
VE     = 3.833030e+02   Tolerance_VE     = 8.206257e-01

h2_A   = 1.588693e-01   Tolerance_h2_A   = 6.350237e-05
h2_D   = 3.401747e-01   Tolerance_h2_D   = 4.654420e-05
h2_AH  = 4.697293e-01   Tolerance_h2_AH  = 4.309939e-05
H2     = 9.687734e-01   Tolerance_H2   = 6.694719e-05
Time for this iteration is : 0 seconds

Iteration: 100
EM-REML
VA     = 1.950874e+03   Tolerance_VA     = 7.694464e-01
VD     = 4.176185e+03   Tolerance_VD     = 5.767536e-01
VAH    = 5.765386e+03   Tolerance_VAH    = 4.911307e-01
VE     = 3.824844e+02   Tolerance_VE     = 8.186132e-01

h2_A   = 1.589316e-01   Tolerance_h2_A   = 6.221256e-05
h2_D   = 3.402207e-01   Tolerance_h2_D   = 4.597600e-05
h2_AH  = 4.696879e-01   Tolerance_h2_AH  = 4.140596e-05
```

```
H2     = 9.688402e-01   Tolerance_H2   = 6.678259e-05
Time for this iteration is : 0 seconds

h2_A: Additive heritability, SE            : 1.589316e-01, 3.424521e-01
h2_D: Dominance heritability, SE           : 3.402207e-01, 4.149391e-01
h2_AH: Haplotype additive heritability, SE : 4.696879e-01, 6.419889e-01
H2: Heritability in the broad sense, SE    : 9.688402e-01, 8.030729e-01

========= end of GREML =========


========= GBLUP begins =========

GBLUP finished!
Time for GBLUP : 0 seconds

Reliabilities of GBLUP finished!
Time for reliabilities : 0 seconds

Calculating marker effects and heritabilities
       reading geno files again
       reading genotype file: snp_geno.dat ...
       genotype file is read
       reading genotype file: snp_geno.dat ...
       genotype file is read

Marker effects and heritabilities finished!

Calculating haplotype heritabilities
       reading hap files again
       reading haplotype file: hap_geno ...
       haplotype file is read
       reading haplotype file: hap_geno1 ...
       haplotype file is read

Haplotype effects and heritabilities finished!
Time for marker (and haplotype) effects and heritabilities: 0 seconds

======= end of GBLUP, time: 0 seconds

========finished reml calc============

Total running time:
0 days 0 hours 0 minutes 1 seconds.
```

5 Analysis of GVCHAP results

5.1 Haplotype and SNP heritability estimates for graphic visualization

GVCHAP calculates and saves SNP effects and heritability estimates and haplotype block heritabilities in a separate file. With the "output_mrk_effect" option in the parameter file, GVCHAP will print additive and marker effects along with SNP position information to 'mrk_eff.snpe' (or user specified) file that can be directly used as the input file for SNPEVEG2. SNPEVG2 is then used to build Manhattan plots and figures by chromosome using the original GBLUP values and identify SNPs and high heritability estimates using SNPEVG2.

Haplotype block heritabilities are printed when the "output_hap_heritabilities" option is specified. To visualize this data alongside SNP heritabilities, the two output files are combined using **merge-mrk-hap.py**. The output of this script can be used directly with SNPEVG2.

merge-mrk-hap.py
Purpose: merges the marker effects output and the haplotype heritabilities
Input: haplotype effects file, marker effects file, hap_block or hap_block_info files, map file
Output: 'snpe' file of all marker and haplotype effects for use with SNPE

The procedure to generate the Manhattan plots and chromosome figures can be summarized as:

1) Open SNPEVG2
2) Load the 'mrk_effect.snpe' file using 'Browse' tab on the GUI of SNPEVG2
3) click 'Setting' and check 'original value' for Y1 axis
4) change 'original value' to user defined title for Y1 axis
5) Click the button pointed by the green arrow to define pixel size and to select color template for the graphs
6) Click 'run'
7) View the graph by scrolling up and down in the top right window
8) Save 'All graphs' or 'Current graph'

The SNPEVG package including SNPEVG2 is available at: http://animalgene.umn.edu/.

5.2 Observed and expected accuracy for predicting phenotypic values

For a validation study, this computing pipeline has a utility program (get-correlations.R) to facilitate calculating the observed accuracy for predicting the phenotypic values using a type of genotypic value (e.g., SNP additive values or haplotype additive values) or a sum of several types of genotypic values (e.g., the sum of SNP dominance values and haplotype additive values) for each validation. This observed accuracy is calculated as the correlation between the predicted genetic values and the phenotypic values in the validation population (individuals flagged as 'V' in the GBLUP output file) that were omitted when calculating GBLUP.

get-correlations.R
Purpose: calculate correlations of GBLUP output and actual phenotypes
Input: phenotype file with original trait and validation column, gblup file,
Output: correlations of predicted and actual phenotypes

Box19: Example of observed accuracy for predicting phenotypic values

```
phenotype.txt  ldl_chol_t    t                 v
gblup_1.out    GBLUP_A       0.899688593648129 0.270864571027161
gblup_1.out    GBLUP_D       0.893025755686799 0.210640618046846
gblup_1.out    GBLUP_AH      0.929131761997618 0.305848907030939
gblup_1.out    GBLUP_G       0.930892023536249 0.307447917595164
```

6 Example for Linux (64-bit)

The following is a short hypothetical example of using the pipeline.

6.1 Data preparation

If starting with SNP data, convert it to VCF format for imputing with BEAGLE. The required input files are the SNP genotype files and a map file. The output is VCF files for each chromosome.

original_geno/chr1

```
ID M_1 M_2 M_3 M_4 M_5 M_6 M_7 M_8 M_9 M_10
Ind_1 0 0 2 0 2 2 2 0 0 2
Ind_2 1 0 2 0 2 2 2 1 2 2
Ind_3 0 0 1 0 2 2 2 0 2 1
Ind_4 2 0 2 0 0 0 0 1 0 2
Ind_5 1 0 2 0 2 2 2 0 2 1
Ind_6 2 0 2 0 0 0 0 0 0 2
Ind_7 0 0 2 0 2 2 2 0 2 2
Ind_8 1 0 1 0 2 2 2 1 2 1
Ind_9 0 0 2 0 2 2 2 0 2 2
Ind_10 2 0 2 0 1 0 1 0 0 2
```

original_geno/chr2

```
ID M_11 M_12 M_13 M_14 M_15 M_16 M_17 M_18 M_19 M_20
Ind_1 0 0 2 2 2 0 2 2 1 2
Ind_2 2 1 0 0 2 2 0 0 0 2
Ind_3 0 0 2 2 2 0 2 2 0 2
Ind_4 0 1 2 2 2 1 2 2 0 1
Ind_5 2 0 0 1 2 2 0 1 1 2
Ind_6 2 0 0 0 2 2 0 0 1 2
Ind_7 0 1 2 2 2 0 2 2 0 1
Ind_8 0 0 2 2 2 1 2 2 0 2
Ind_9 0 0 2 2 2 0 2 2 0 2
Ind_10 2 1 0 0 2 2 0 0 0 1
```

```
./convert-to-vcf.py -i original_geno/chr1 original_geno/chr2 -m map_data.txt -o original_geno
```

original_geno_1.vcf

```
##fileformat=VCFv4.2
##fileDate=2019-11-13-09:27
##source=GVCHAP-converter
##FORMAT=<ID=GT,Number=1,Type=String,Description="Genotype">
#CHROM POS ID REF ALT QUAL FILTER INFO FORMAT Ind_1 Ind_2 Ind_3 Ind_4 Ind_5 Ind_6 Ind_7 Ind_8 Ind_9 Ind_10
1 485953  M_1  T C - - - GT 0/0 0/1 0/0 1/1 0/1 1/1 0/0 0/1 0/0 1/1
1 1666173 M_2  T C - - - GT 0/0 0/0 0/0 0/0 0/0 0/0 0/0 0/0 0/0 0/0
1 2753327 M_3  T C - - - GT 1/1 1/1 0/1 1/1 1/1 1/1 1/1 0/1 1/1 1/1
1 3078783 M_4  T C - - - GT 0/0 0/0 0/0 0/0 0/0 0/0 0/0 0/0 0/0 0/0
1 3186152 M_5  T C - - - GT 1/1 1/1 1/1 0/0 1/1 0/0 1/1 1/1 1/1 0/1
1 3186260 M_6  T C - - - GT 1/1 1/1 1/1 0/0 1/1 0/0 1/1 1/1 1/1 0/0
1 3270679 M_7  T C - - - GT 1/1 1/1 1/1 0/0 1/1 0/0 1/1 1/1 1/1 0/1
```

```
1 3830498 M_8  T C - - - GT 0/0 0/1 0/0 0/1 0/0 0/0 0/0 0/1 0/0 0/0
1 4609477 M_9  T C - - - GT 0/0 1/1 1/1 0/0 1/1 0/0 1/1 1/1 1/1 0/0
1 4764811 M_10 T C - - - GT 1/1 1/1 0/1 1/1 0/1 1/1 1/1 0/1 1/1 1/1
```

original_geno_2.vcf

```
##fileformat=VCFv4.2
##fileDate=2019-11-13-09:27
##source=GVCHAP-converter
##FORMAT=<ID=GT,Number=1,Type=String,Description="Genotype">
#CHROM POS ID REF ALT QUAL FILTER INFO FORMAT Ind_1 Ind_2 Ind_3 Ind_4 Ind_5 Ind_6 Ind_7
Ind_8 Ind_9 Ind_10
2 730589  M_11 T C - - - GT 0/0 1/1 0/0 0/0 1/1 1/1 0/0 0/0 0/0 1/1
2 755444  M_12 T C - - - GT 0/0 0/1 0/0 0/1 0/0 0/0 0/1 0/0 0/0 0/1
2 841463  M_13 T C - - - GT 1/1 0/0 1/1 1/1 0/0 0/0 1/1 1/1 1/1 0/0
2 841675  M_14 T C - - - GT 1/1 0/0 1/1 1/1 0/1 0/0 1/1 1/1 1/1 0/0
2 851161  M_15 T C - - - GT 1/1 1/1 1/1 1/1 1/1 1/1 1/1 1/1 1/1 1/1
2 1281122 M_16 T C - - - GT 0/0 1/1 0/0 0/1 1/1 1/1 0/0 0/1 0/0 1/1
2 1360754 M_17 T C - - - GT 1/1 0/0 1/1 1/1 0/0 0/0 1/1 1/1 1/1 0/0
2 1723802 M_18 T C - - - GT 1/1 0/0 1/1 1/1 0/1 0/0 1/1 1/1 1/1 0/0
2 1824359 M_19 T C - - - GT 0/1 0/0 0/0 0/0 0/1 0/1 0/0 0/0 0/0 0/0
2 1841620 M_20 T C - - - GT 1/1 1/1 1/1 0/1 1/1 1/1 0/1 1/1 1/1 0/1
```

Impute the VCF files with BEAGLE. Running the imputation for each chromosome separately may be advantageous for large data.

```
java –Xmx8g -jar beagle.27Jan18.7e1.jar gt=original_geno_1.vcf nthreads=4 window=600 overlap=60 out=imputed_geno_chr1

java –Xmx8g -jar path/to/beagle.27Jan18.7e1.jar gt=original_geno_2.vcf nthreads=4 window=600 overlap=60 out=imputed_geno_chr2
```

Setting RAM limit and number of threads is not necessary for small data. Without these parameters and using the default options for BEAGLE the command for the first chromosome is simpler.

```
java beagle.27Jan18.7e1.jar gt=original_geno_1.vcf out=imputed_geno_chr1
```

This will output one imputed VCF file per chromosome compressed via gzip.

Extract the imputed VCF file.

```
gunzip imputed_geno_chr*
```

imputed_geno_chr1.vcf

```
##fileformat=VCFv4.2
##filedate=20200226
##source="beagle.27Jan18.7e1.jar (version 4.1)"
##INFO=<ID=AF,Number=A,Type=Float,Description="Estimated ALT Allele Frequencies">
##INFO=<ID=AR2,Number=1,Type=Float,Description="Allelic R-Squared: estimated squared correlation between most probable REF dose and true REF dose">
##INFO=<ID=DR2,Number=1,Type=Float,Description="Dosage R-Squared: estimated squared correlation between estimated REF dose [P(RA) + 2*P(RR)] and true REF dose">
##INFO=<ID=IMP,Number=0,Type=Flag,Description="Imputed marker">
```

```
##FORMAT=<ID=GT,Number=1,Type=String,Description="Genotype">
##FORMAT=<ID=DS,Number=A,Type=Float,Description="estimated ALT dose [P(RA) + P(AA)]">
##FORMAT=<ID=GP,Number=G,Type=Float,Description="Estimated Genotype Probability">
#CHROM POS ID REF ALT QUAL FILTER INFO FORMAT Ind_1 Ind_2 Ind_3 Ind_4 Ind_5 Ind_6 Ind_7
Ind_8 Ind_9 Ind_10
1 485953  M_1  T C . PASS . GT 0|0 1|0 0|0 1|1 0|1 1|1 0|0 1|0 0|0 1|1
1 1666173 M_2  T C . PASS . GT 0|0 0|0 0|0 0|0 0|0 0|0 0|0 0|0 0|0 0|0
1 2753327 M_3  T C . PASS . GT 1|1 1|1 0|1 1|1 1|1 1|1 1|1 1|0 1|1 1|1
1 3078783 M_4  T C . PASS . GT 0|0 0|0 0|0 0|0 0|0 0|0 0|0 0|0 0|0 0|0
1 3186152 M_5  T C . PASS . GT 1|1 1|1 1|1 0|0 1|1 0|0 1|1 1|1 1|1 0|1
1 3186260 M_6  T C . PASS . GT 1|1 1|1 1|1 0|0 1|1 0|0 1|1 1|1 1|1 0|0
1 3270679 M_7  T C . PASS . GT 1|1 1|1 1|1 0|0 1|1 0|0 1|1 1|1 1|1 0|1
1 3830498 M_8  T C . PASS . GT 0|0 1|0 0|0 0|1 0|0 0|0 0|0 1|0 0|0 0|0
1 4609477 M_9  T C . PASS . GT 0|0 1|1 1|1 0|0 1|1 0|0 1|1 1|1 1|1 0|0
1 4764811 M_10 T C . PASS . GT 1|1 1|1 1|0 1|1 1|0 1|1 1|1 1|0 1|1 1|1
```

imputed_geno_chr2.vcf

```
##fileformat=VCFv4.2
##filedate=20200226
##source="beagle.27Jan18.7e1.jar (version 4.1)"
##INFO=<ID=AF,Number=A,Type=Float,Description="Estimated ALT Allele Frequencies">
##INFO=<ID=AR2,Number=1,Type=Float,Description="Allelic R-Squared: estimated squared
correlation between most probable REF dose and true REF dose">
##INFO=<ID=DR2,Number=1,Type=Float,Description="Dosage R-Squared: estimated squared
correlation between estimated REF dose [P(RA) + 2*P(RR)] and true REF dose">
##INFO=<ID=IMP,Number=0,Type=Flag,Description="Imputed marker">
##FORMAT=<ID=GT,Number=1,Type=String,Description="Genotype">
##FORMAT=<ID=DS,Number=A,Type=Float,Description="estimated ALT dose [P(RA) + P(AA)]">
##FORMAT=<ID=GP,Number=G,Type=Float,Description="Estimated Genotype Probability">
#CHROM POS ID REF ALT QUAL FILTER INFO FORMAT Ind_1 Ind_2 Ind_3 Ind_4 Ind_5 Ind_6 Ind_7
Ind_8 Ind_9 Ind_10
2 730589  M_11 T C . PASS . GT 0|0 1|1 0|0 0|0 1|1 1|1 0|0 0|0 0|0 1|1
2 755444  M_12 T C . PASS . GT 0|0 1|0 0|0 0|1 0|0 0|0 0|1 0|0 0|0 1|0
2 841463  M_13 T C . PASS . GT 1|1 0|0 1|1 1|1 0|0 0|0 1|1 1|1 1|1 0|0
2 841675  M_14 T C . PASS . GT 1|1 0|0 1|1 1|1 0|1 0|0 1|1 1|1 1|1 0|0
2 851161  M_15 T C . PASS . GT 1|1 1|1 1|1 1|1 1|1 1|1 1|1 1|1 1|1 1|1
2 1281122 M_16 T C . PASS . GT 0|0 1|1 0|0 0|1 1|1 1|1 0|0 1|0 0|0 1|1
2 1360754 M_17 T C . PASS . GT 1|1 0|0 1|1 1|1 0|0 0|0 1|1 1|1 1|1 0|0
2 1723802 M_18 T C . PASS . GT 1|1 0|0 1|1 1|1 0|1 0|0 1|1 1|1 1|1 0|0
```

Convert the imputed haplotypes from VCF. A VCF with all chromosomes merged may be used, the program will separate them. Output haplotypes files will, by default, be in “hap” folder, SNP genotype files in “geno” folder, one file per chromosome. The map can be extracted from VCF files via the “--map” option.

```
./convert-from-vcf.py -i imputed_geno_chr1.vcf imputed_geno_chr2.vcf --map
map.txt
```

hap/chr1

```
Ind_1  1 1 1 1 2 2 1 1 2 2 2 2 2 2 1 1 1 1 2 2
Ind_2  2 1 1 1 2 2 1 1 2 2 2 2 2 2 2 1 2 2 2 2
Ind_3  1 1 1 1 1 2 1 1 2 2 2 2 2 2 1 1 2 2 2 1
Ind_4  2 2 1 1 2 2 1 1 1 1 1 1 1 1 1 2 1 1 2 2
Ind_5  1 2 1 1 2 2 1 1 2 2 2 2 2 2 1 1 2 2 2 1
```

```
Ind_6  2 2 1 1 2 2 1 1 1 1 1 1 1 1 1 1 1 1 2 2
Ind_7  1 1 1 1 2 2 1 1 2 2 2 2 2 2 1 1 2 2 2 2
Ind_8  2 1 1 1 2 1 1 1 2 2 2 2 2 2 2 1 2 2 2 1
Ind_9  1 1 1 1 2 2 1 1 2 2 2 2 2 2 1 1 2 2 2 2
Ind_10 2 2 1 1 2 2 1 1 1 2 1 1 1 2 1 1 1 1 2 2
```

hap/chr2

```
Ind_1  1 1 1 1 2 2 2 2 2 2 1 1 2 2 2 2
Ind_2  2 2 2 1 1 1 1 1 2 2 2 2 1 1 1 1
Ind_3  1 1 1 1 2 2 2 2 2 2 1 1 2 2 2 2
Ind_4  1 1 1 2 2 2 2 2 2 2 1 2 2 2 2 2
Ind_5  2 2 1 1 1 1 1 2 2 2 2 2 1 1 1 2
Ind_6  2 2 1 1 1 1 1 1 2 2 2 2 1 1 1 1
Ind_7  1 1 1 2 2 2 2 2 2 2 1 1 2 2 2 2
Ind_8  1 1 1 1 2 2 2 2 2 2 2 1 2 2 2 2
Ind_9  1 1 1 1 2 2 2 2 2 2 1 1 2 2 2 2
Ind_10 2 2 2 1 1 1 1 1 2 2 2 2 1 1 1 1
```

map.txt

```
SNPID  Chr    Position
M_1    1      485953
M_2    1      1666173
M_3    1      2753327
M_4    1      3078783
M_5    1      3186152
M_6    1      3186260
M_7    1      3270679
M_8    1      3830498
M_9    1      4609477
M_10   1      4764811
M_11   2      730589
M_12   2      755444
M_13   2      841463
M_14   2      841675
M_15   2      851161
M_16   2      1281122
M_17   2      1360754
M_18   2      1723802
M_19   2      1824359
M_20   2      1841620
```

Define haplotype blocks. Here we create the hap_info files for blocks of 4 SNPs. Since there are 10 SNPs, we have 2 full blocks and the last block is left with 2 SNPs for 3 blocks total.

```
./block-by-snp.py -i hap/chr1 hap/chr2 --snp 4 -o hap_info_snp_4
```

hap_info_snp_4/hap_block_1

```
blk1   0 3
blk2   4 7
blk3   8 10
```

hap_info_snp_4/hap_block_1

```
blk4   0 3
```

```
blk5    4 7
blk6    8 10
```

Using the hap_info files from the last step and the haplotype files create the 4 SNP block haplotype genotype files.

```
./get-hap-geno.py --hap hap/chr1 hap/chr2 --hapinfo hap_info_snp_4/hap_block_1 hap_info_snp_4/hap_block_2 -o hap_geno_snp_4
```

hap_geno_snop_4/hap_geno_1

```
ID      hap_1_1       hap_1_1       hap_1_2       hap_1_2       hap_1_3       hap_1_3
Ind_1   1      1      1      1      1      1
Ind_2   2      1      2      1      2      2
Ind_3   3      1      1      1      2      3
Ind_4   2      2      3      4      1      1
Ind_5   1      2      1      1      2      3
Ind_6   2      2      3      3      1      1
Ind_7   1      1      1      1      2      2
Ind_8   2      3      2      1      2      3
Ind_9   1      1      1      1      2      2
Ind_10  2      2      3      5      1      1
```

hap_geno_snop_4/hap_geno_2

```
ID      hap_2_1       hap_2_1       hap_2_2       hap_2_2
Ind_1   1      1      1      1
Ind_2   2      3      2      2
Ind_3   1      1      1      1
Ind_4   1      4      1      3
Ind_5   3      5      2      4
Ind_6   3      3      2      2
Ind_7   1      4      1      1
Ind_8   1      1      3      1
Ind_9   1      1      1      1
Ind_10  2      3      2      2
```

The phenotype file is provided by the user, the following is an example.

phenotypes.txt

```
sample_id      factor1  factor2   cov1     cov2     trait1   trait1_val
Ind_1          1        B         1.1      4        1.7      1.7
Ind_2          1        A         2.3      3        1.2      -9999
Ind_3          2        B         0.7      8        1.3      1.3
Ind_4          2        B         0.3      2        2.1      2.1
Ind_5          1        A         3.5      5        2.3      -9999
Ind_6          2        A         1.5      9        0.3      0.3
Ind_8          3        B         2.8      7        1.5      1.5
Ind_9          3        A         3.6      4        3.8      -9999
Ind_10         2        A         5.1      8        3.0      3.0
```

6.2 Configuring and running GVCHAP

Copy the parameter file template and edit the options using any text editor.

```
cp parameter_template.txt parameter.txt
```

Generate the list of SNP and haplotype genotype files and add them to the parameter file. Each program prints a list of specified chromosome files with the prefix, number of SNPs or haplotype blocks and relative file path to standard out. This output can be redirected to append a file (parameter.txt) using ">>". It is advised to run the program from the same directory GVCHAP will launch as the paths printed are relative.

```
./count-snps.py -i geno/chr1 geno/chr2 >> parameter.txt

./count-haps.py -i hap_geno_snp_4/hap_geno_1 hap_geno_snp_4/hap_geno_2 >>
parameter.txt
```

parameter.txt

```
num_iter 100
var_snp_a 1
var_snp_d 2
var_snp_e 3
var_hap_a 5
tolerance_var 1.0E-08
tolerance_heritability 1.0E-06
traits_col 7
#factors_pos 2 3
#covar_pos 2 3
num_ind 10
missing_phen_val -9999
missing_hap_val -9999
use_ai_reml 3
out_greml ./out/greml.txt
out_gblup ./out/gblup.txt
output_fixed_effect ./out/fixed_effect.txt
output_hap_heritabilities ./out/hap_heritabilites.snpe
output_mrk_effect ./out/mrk_effect.snpe
out_rel  ./out/relationship.txt
#save_g test
#load_g test
map_file map_new.txt
phenotype phenotypes.txt
geno_snp 10 geno/chr1
geno_snp 10 geno/chr2
geno_hap 3 hap_geno_snp_4/hap_geno_1
geno_hap 3 hap_geno_snp_4/hap_geno_2
```

Set the preferred number of threads. For small data, use a lower number to reduce overhead. The maximum number of threads should not exceed the number of processor cores.

```
export OMP_NUM_THREADS=4
```

Create the output directory and run GVCHAP.

```
mkdir out

./gvchap parameter.txt
```

6.3 GVCHAP results

greml.txt (truncated)

```
Output of GREML results from GVCHAP

Number of markers: 18
Number of chromosomes: 2

Number of phenotypic observations: 6
  Number of individuals with phenotypic observations: 6
  Number of individuals without phenotypic observations: 4

Iteration VA             Tolerance_VA   ...  VE             Tolerance_VE
1         4.678770e-02   9.532123e-01   ...  4.916993e-01   2.508301e+00
2         1.447511e-02   3.231259e-02   ...  5.572170e-01   6.551770e-02
3         4.157133e-03   1.031798e-02   ...  6.174821e-01   6.026512e-02
.                                       ...
.                                       ...
.                                       ...
50        1.436573e-42   9.160369e-42   ...  7.989982e-01   4.797080e-07
51        1.947485e-43   1.241825e-42   ...  7.989986e-01   3.772796e-07
52        2.640096e-44   1.683475e-43   ...  7.989989e-01   2.967221e-07
SE        2.078643e+00                  ...  2.199821e+00

Inverse of AI matrix:
   4.320758e+00   1.847348e+00   1.635757e-01  -1.679783e+00
   1.847348e+00   3.140276e+00   3.397000e+00  -3.307465e+00
   1.635757e-01   3.397000e+00   1.020816e+01  -6.162503e+00
  -1.679783e+00  -3.307465e+00  -6.162503e+00   4.839214e+00

h2_A: Additive heritability, SE           : 3.304245e-44, 2.601552e+00
h2_D: Dominance heritability, SE          : 3.112909e-06, 2.217860e+00
h2_AH: Haplotype additive heritability, SE : 1.556136e-22, 3.998768e+00
H2: Heritability in the broad sense, SE : 3.112909e-06, 6.679748e+00
```

gblup.txt (part 1/2)

```
ID        GBLUP_A          Reliability_A    GBLUP_D          Reliability_D
Ind_1     -1.417076e-45    7.940325e-44     -3.002388e-07    2.374568e-06
Ind_2     3.417581e-45     5.398822e-44     -2.673550e-07    1.597983e-06
Ind_3     -1.141988e-44    1.323107e-43     -4.469567e-07    2.316568e-06
Ind_4     1.158666e-44     3.762606e-44     -2.528310e-06    3.916813e-06
Ind_5     -5.251502e-45    3.708986e-44     -6.316190e-07    1.773615e-07
Ind_6     5.584960e-45     1.407367e-43     -3.150597e-06    3.599992e-06
Ind_7     -2.083943e-45    1.140496e-43     -8.977509e-08    2.785376e-06
Ind_8     -6.918614e-45    1.078317e-43     -3.209811e-07    8.796044e-07
Ind_9     -8.085614e-45    1.258761e-43     -3.811866e-07    2.869172e-06
Ind_10    1.458743e-44     1.422649e-43     1.921770e-06     2.774361e-06
```

gblup.txt (part 2/2)

GBLUP_AH	Reliability_AH	GBLUP_G	Reliability_G	Train./Valid.
5.495068e-23	1.821642e-22	-3.002388e-07	2.374568e-06	T
-7.675393e-24	9.878306e-23	-2.673550e-07	1.597983e-06	V
-3.569221e-23	4.032895e-22	-4.469567e-07	2.316568e-06	T
8.131985e-23	1.568144e-22	-2.528310e-06	3.916813e-06	T
-6.370900e-23	5.786434e-23	-6.316190e-07	1.773615e-07	V
-7.689294e-23	4.717664e-22	-3.150597e-06	3.599992e-06	T
3.861063e-24	2.406457e-22	-8.977509e-08	2.785376e-06	V
-2.250774e-23	3.184484e-22	-3.209811e-07	8.796044e-07	T
-1.097145e-23	2.939961e-22	-3.811866e-07	2.869172e-06	V
8.626368e-23	4.217356e-22	1.921770e-06	2.774361e-06	T

mrk_effect.txt (part 1/4)

SNPID	Chr	Position	Effect_A	m_effect_A	Effect_D	m_effect_D
M_1	1	485953	-8.225998e-07	-1.480680e-05	-9.582880e-55	-1.724918e-53
M_2	1	1666173	0.000000e+00	0.000000e+00	0.000000e+00	0.000000e+00
M_3	1	2753327	-7.914630e-07	-1.424633e-05	-1.800289e-55	-3.240521e-54
M_4	1	3078783	0.000000e+00	0.000000e+00	0.000000e+00	0.000000e+00
M_5	1	3186152	8.148142e-07	1.466666e-05	4.102357e-55	7.384242e-54
M_6	1	3186260	1.582926e-06	2.849267e-05	-7.201158e-55	-1.296208e-53
M_7	1	3270679	8.148142e-07	1.466666e-05	4.102357e-55	7.384242e-54
M_8	1	3830498	7.291893e-07	1.312541e-05	-2.487959e-55	-4.478327e-54
M_9	1	4609477	3.103578e-06	5.586441e-05	-7.059508e-55	-1.270711e-53
M_10	1	4764811	-7.914630e-07	-1.424633e-05	-2.700434e-55	-4.860782e-54
M_11	2	730589	1.462231e-19	2.632016e-18	-1.872968e-67	-3.371343e-66
M_12	2	755444	-3.892243e-08	-7.006037e-07	1.770686e-56	3.187235e-55
M_13	2	841463	-1.462231e-19	-2.632016e-18	-1.872968e-67	-3.371343e-66
M_14	2	841675	-1.060355e-19	-1.908639e-18	-1.730581e-67	-3.115046e-66
M_15	2	851161	0.000000e+00	0.000000e+00	0.000000e+00	0.000000e+00
M_16	2	1281122	-3.113684e-08	-5.604631e-07	3.541248e-56	6.374246e-55
M_17	2	1360754	-1.462231e-19	-2.632016e-18	-1.872968e-67	-3.371343e-66
M_18	2	1723802	-1.060355e-19	-1.908639e-18	-1.730581e-67	-3.115046e-66

mrk_effect.txt (part 2/4)

Effect_A2	m_effect_A2	Effect_D2	m_effect_D2	h2_mrk_A
8.225998e-07	1.480680e-05	9.582880e-55	1.724918e-53	3.079715e-07
0.000000e+00	0.000000e+00	0.000000e+00	0.000000e+00	0.000000e+00
7.914630e-07	1.424633e-05	1.800289e-55	3.240521e-54	2.850983e-07
0.000000e+00	0.000000e+00	0.000000e+00	0.000000e+00	0.000000e+00
8.148142e-07	1.466666e-05	4.102357e-55	7.384242e-54	3.021695e-07
1.582926e-06	2.849267e-05	7.201158e-55	1.296208e-53	1.140393e-06
8.148142e-07	1.466666e-05	4.102357e-55	7.384242e-54	3.021695e-07
7.291893e-07	1.312541e-05	2.487959e-55	4.478327e-54	2.419992e-07
3.103578e-06	5.586441e-05	7.059508e-55	1.270711e-53	4.383881e-06
7.914630e-07	1.424633e-05	2.700434e-55	4.860782e-54	2.850983e-07
1.462231e-19	2.632016e-18	1.872968e-67	3.371343e-66	9.731179e-33
3.892243e-08	7.006037e-07	1.770686e-56	3.187235e-55	6.894982e-10
1.462231e-19	2.632016e-18	1.872968e-67	3.371343e-66	9.731179e-33
1.060355e-19	1.908639e-18	1.730581e-67	3.115046e-66	5.117244e-33
0.000000e+00	0.000000e+00	0.000000e+00	0.000000e+00	0.000000e+00
3.113684e-08	5.604631e-07	3.541248e-56	6.374246e-55	4.412477e-10
1.462231e-19	2.632016e-18	1.872968e-67	3.371343e-66	9.731179e-33
1.060355e-19	1.908639e-18	1.730581e-67	3.115046e-66	5.117244e-33

mrk_effect.txt (part 3/4)

m_h2_mrk_A	h2_mrk_D	m_h2_mrk_D	H2_mrk	h2_mrk_std_A
5.543488e-06	1.728595e-54	3.111471e-53	3.079715e-07	-9.454577e-02
0.000000e+00	0.000000e+00	0.000000e+00	0.000000e+00	-4.016872e-01
5.131769e-06	6.100787e-56	1.098142e-54	2.850983e-07	-1.173574e-01
0.000000e+00	0.000000e+00	0.000000e+00	0.000000e+00	-4.016872e-01
5.439050e-06	3.167875e-55	5.702174e-54	3.021695e-07	-1.003322e-01
2.052707e-05	9.761258e-55	1.757027e-53	1.140393e-06	7.356321e-01
5.439050e-06	3.167875e-55	5.702174e-54	3.021695e-07	-1.003322e-01
4.355986e-06	1.165166e-55	2.097298e-54	2.419992e-07	-1.603403e-01
7.890986e-05	9.381019e-55	1.688583e-53	4.383881e-06	3.970378e+00
5.131769e-06	1.372677e-55	2.470819e-54	2.850983e-07	-1.173574e-01
1.751612e-31	6.603314e-80	1.188596e-78	9.731179e-33	-4.016872e-01
1.241097e-08	5.901799e-58	1.062324e-56	6.894982e-10	-4.009996e-01
1.751612e-31	6.603314e-80	1.188596e-78	9.731179e-33	-4.016872e-01
9.211039e-32	5.637482e-80	1.014747e-78	5.117244e-33	-4.016872e-01
0.000000e+00	0.000000e+00	0.000000e+00	0.000000e+00	-4.016872e-01
7.942459e-09	2.360553e-57	4.248995e-56	4.412477e-10	-4.012472e-01
1.751612e-31	6.603314e-80	1.188596e-78	9.731179e-33	-4.016872e-01
9.211039e-32	5.637482e-80	1.014747e-78	5.117244e-33	-4.016872e-01

mrk_effect.txt (part 4/4)

m_h2_mrk_std_A	h2_mrk_std_D	m_h2_mrk_std_D	H2_mrk_std
-1.701824e+00	2.281714e-20	4.107085e-19	-9.454577e-02
-7.230370e+00	-3.952598e-21	-7.114676e-20	-4.016872e-01
-2.112433e+00	-3.007805e-21	-5.414048e-20	-1.173574e-01
-7.230370e+00	-3.952598e-21	-7.114676e-20	-4.016872e-01
-1.805980e+00	9.533040e-22	1.715947e-20	-1.003322e-01
1.324138e+01	1.116409e-20	2.009536e-19	7.356321e-01
-1.805980e+00	9.533040e-22	1.715947e-20	-1.003322e-01
-2.886125e+00	-2.148174e-21	-3.866713e-20	-1.603403e-01
7.146680e+01	1.057524e-20	1.903542e-19	3.970378e+00
-2.112433e+00	-1.826813e-21	-3.288264e-20	-1.173574e-01
-7.230370e+00	-3.952598e-21	-7.114676e-20	-4.016872e-01
-7.217992e+00	-3.943458e-21	-7.098224e-20	-4.009996e-01
-7.230370e+00	-3.952598e-21	-7.114676e-20	-4.016872e-01
-7.230370e+00	-3.952598e-21	-7.114676e-20	-4.016872e-01
-7.230370e+00	-3.952598e-21	-7.114676e-20	-4.016872e-01
-7.222449e+00	-3.916041e-21	-7.048874e-20	-4.012472e-01
-7.230370e+00	-3.952598e-21	-7.114676e-20	-4.016872e-01
-7.230370e+00	-3.952598e-21	-7.114676e-20	-4.016872e-01

hap_heritabilities.txt

HAPID	h2_hap_AH	H2_hap	h2_hap_std_AH	H2_hap_std
0	9.763077e-02	9.763077e-02	-3.539883e-01	-3.539883e-01
1	1.336033e-01	1.336033e-01	-2.295952e-01	-2.295952e-01
2	7.686044e-01	7.686044e-01	1.966242e+00	1.966242e+00
3	8.172086e-05	8.172086e-05	-6.913134e-01	-6.913134e-01
4	7.263791e-05	7.263791e-05	-6.913448e-01	-6.913448e-01

fixed_effect.txt

Fixed_effect	Level_name	Level	Value
0	mu	1	1.650001e+00

6.4 Analysis of GVCHAP results

To visualize marker effects and marker and haplotype heritabilities using SNPEVG2, merge the mrk_effect.snpe and hap_heritabilities.snpe files using the following script.

```
	./merge-mrk-hap.py -sh out/mrk_effect.txt -hh out/hap_heritabilites.txt --
hapinfo hap_info_snp_4/hap_block_info_* --map map.txt -o all.snpe
```

all.snpe (truncated)

```
SNPID Chr Position Effect_A      ...  h2_hap_AH    H2_hap       ...
M_1   1   485953  -8.225998e-07 ...  9.763077e-02 9.763077e-02 ...
M_2   1   1666173  0.000000e+00 ...  9.763077e-02 9.763077e-02 ...
M_3   1   2753327 -7.914630e-07 ...  9.763077e-02 9.763077e-02 ...
M_4   1   3078783  0.000000e+00 ...  9.763077e-02 9.763077e-02 ...
M_5   1   3186152  8.148142e-07 ...  1.336033e-01 1.336033e-01 ...
M_6   1   3186260  1.582926e-06 ...  1.336033e-01 1.336033e-01 ...
M_7   1   3270679  8.148142e-07 ...  1.336033e-01 1.336033e-01 ...
M_8   1   3830498  7.291893e-07 ...  1.336033e-01 1.336033e-01 ...
M_9   1   4609477  3.103578e-06 ...  7.686044e-01 7.686044e-01 ...
M_10  1   4764811 -7.914630e-07 ...  7.686044e-01 7.686044e-01 ...
M_11  2   730589   1.462231e-19 ...  8.172086e-05 8.172086e-05 ...
M_12  2   755444  -3.892243e-08 ...  8.172086e-05 8.172086e-05 ...
M_13  2   841463  -1.462231e-19 ...  8.172086e-05 8.172086e-05 ...
M_14  2   841675  -1.060355e-19 ...  8.172086e-05 8.172086e-05 ...
M_15  2   851161   0.000000e+00 ...  7.263791e-05 7.263791e-05 ...
M_16  2   1281122 -3.113684e-08 ...  7.263791e-05 7.263791e-05 ...
M_17  2   1360754 -1.462231e-19 ...  7.263791e-05 7.263791e-05 ...
M_18  2   1723802 -1.060355e-19 ...  7.263791e-05 7.263791e-05 ...
```

To calculate the Pearson correlation between the predicted values in gblup.out and the known phenotype (trait1), run the following script.

```
	./get-correlations.R -p phenotypes.txt -i sample_id --gblup out/gblup.out -t
trait1  -v trait1_val -m -9999 -b GBLUP_A,GBLUP_D,GBLUP_AH,GBLUP_G
```

The example below is from a real dataset, not from the hypothetical example as all results above. The output is one line for each of the BLUP values. The first numerical value is the correlation of the training population (T values in the gblup file). The second numerical value is the correlation of the validation population (V values in the blup file).

```
phenotype.txt  ldl_chol_t    t                 v
gblup_1.out    GBLUP_A       0.899688593648129 0.270864571027161
gblup_1.out    GBLUP_D       0.893025755686799 0.210640618046846
gblup_1.out    GBLUP_AH      0.929131761997618 0.305848907030939
gblup_1.out    GBLUP_G       0.930892023536249 0.307447917595164
```

7 Example for Windows 10

This following summary shows the slight difference in commands when using the pipeline in Windows 10 PowerShell. The order of commands follows the above Example for Linux. Make sure the python and Rscript executables are in your PATH.

7.1 Data preparation

```
python.exe ..\convert-to-vcf.py -i .\original_geno\chr1 .\original_geno\chr2 --map
.\map_data.txt -o original_geno

python.exe .\convert-from-vcf.py --map .\map.txt -i .\imputed_geno_chr1.vcf
.\imputed_geno_chr2.vcf

python.exe .\block-by-snp.py -i .\hap\chr1 .\hap\chr2 --snp 4 -o .\hap_info_snp_4

python.exe .\get-hap-geno.py --hap .\hap\chr1 .\hap\chr2 --hapinfo
.\hap_info_snp_4\hap_block_1 .\hap_info_snp_4\hap_block_2 -o hap_geno_snp_4
```

7.2 Configuring and running GVCHAP

```
cp parameter_template.txt parameter.txt

python.exe .\count-snps.py -i .\geno\chr1 .\geno\chr2

python.exe .\count-haps.py -i .\hap_geno_snp_4\hap_geno_1 .\hap_geno_snp_4\hap_geno_2
```

You will have to manually add the output of the above two commands to your parameter file.

```
set OMP_NUM_THREADS=4

mkdir out

.\gvchap.exe .\parameter.txt
```

7.3 Analysis of GVCHAP results

```
python.exe .\merge-mrk-hap.py -sh .\out\mrk_effect.snpe -hh
.\out\hap_heritabilites.snpe --hapinfo .\hap_info_snp_4\hap_block_info_1
.\hap_info_snp_4\hap_block_info_2 --map .\map.txt -o all.snpe

Rscript.exe .\get-correlations.R -p .\phenotypes.txt -i sample_id --gblup
.\out\gblup.txt -t trait1 -v trait1_val -m -9999 -b GBLUP_A,GBLUP_D,GBLUP_AH,GBLUP_G
```

8 Multi-node Processing (MNP) of Genomic Relationship Matrices

The MNP program is the multi-node implementation of the two-step strategy to save and read genomic relationship matrices for repeated runs using the same relationship matrices, such as cross validations and multi-trait analysis. MNP is able to process large datasets that cannot be processed using a single node. The output of this program are binary files that can be loaded by GVCHAP. This program currently is available for Linux only.

8.1 Command line options

The MNP program has a built-in help information to explain the commands. Typing

```
./MNP -h
```
or
```
./MNP --help
```

will display the following options for required parapeters:

```
-s, --step        The step of the MNP execution that specifies what to do using
                  MNP. One of the Three choices must be selected:
                     split_big_file: split the original SNP or haplotype files into
                     small files
                     sub_ww: the WW' for a small file
                     grm: genomic relationship matrix
-f, --ftype       The type of input file. One of two choices must be selected:
                     'hap' for haplotype genotypes or 'geno' for SNP genotypes
-i, --input       The input file name required for all but 'grm' steps
-w, --width       Only for 'split_big_file' step, this option sets the number of
                  SNPs or haplotype blocks to split the input file by [default:200]
-t, --threads     The number of processor threads to use. Suggested limiting number
                  is the number of cores not the number of logical processors.
-o, --output      The prefix of output file name(s) [default: input file name]
                  [required for 'grm' step]
```

8.2 MNP for SNP additive and dominance genomic relationship matrices

8.2.1 Split SNP genotype files into small files

The first step splits each chromosome by user defined number of SNPs. Assume two chromosomes 'geno_chr1.dat' and 'geno_chr2.dat' with 1,800 and 400 SNPs respectively. The following will split each chromosome into small files each with 500 SNPs.

```
./MNP -s split_big_file -i geno_chr1.dat -f geno -w 500 -o geno_sub_chr1
./MNP -s split_big_file -i geno_chr2.dat -f geno -w 500 -o geno_sub_chr2
```

The input files are split into smaller text files in the directory the program is executed from. The files will be named according to the specified prefix '-o' with underscore and part number at the end. For a chromosome with fewer than the number SNPs specified by option '–w' (500 in this example) the MNP program copies the original file to the path generated by this run as it cannot be split. This step will output the following smaller files:

```
geno_sub_chr1_1
geno_sub_chr1_2
geno_sub_chr1_3
geno_sub_chr1_4
geno_sub_chr2_1
```

In the above files, the first 3 files each has 500 SNPs, the 4th has 300 SNPs, and the 5th has 400 SNPs. These files are saved as text files.

8.2.2 Calculate $\mathbf{W}_\alpha^i \mathbf{W}_\alpha^i{}'$ and $\mathbf{W}_\delta^i \mathbf{W}_\delta^i{}'$ for each split file

This step calculates $\mathbf{W}_\alpha^i \mathbf{W}_\alpha^i{}'$ and $\mathbf{W}_\delta^i \mathbf{W}_\delta^i{}'$ for each of the split SNP genotype files. The command is:

```
./MNP -s sub_ww -f geno -i geno_sub_chr1_1 -p 4
./MNP -s sub_ww -f geno -i geno_sub_chr1_2 -p 4
./MNP -s sub_ww -f geno -i geno_sub_chr1_3 -p 4
./MNP -s sub_ww -f geno -i geno_sub_chr1_4 -p 4
./MNP -s sub_ww -f geno -i geno_sub_chr2_1 -p 4
```

The $\mathbf{W}_\alpha^i \mathbf{W}_\alpha^i{}'$ matrices are saved in temporary folder 'tmp_geno_a_ww_split_matrix' folder, and $\mathbf{W}_\delta^i \mathbf{W}_\delta^i{}'$ matrices are saved in 'tmp_geno_d_ww_split_matrix'. These two folders are temporary and are deleted after the calculations of the next step.

8.2.3 Calculate SNP genomic relationship matrix and save as binary files

The MNP program will automatically use the saved $\mathbf{W}_\alpha^i \mathbf{W}_\alpha^i{}'$ and $\mathbf{W}_\delta^i \mathbf{W}_\delta^i{}'$ matrices for calculation of the genomic relationship matrices using the following formulae:

$$\mathbf{A}_g = \mathbf{W}_\alpha \mathbf{W}_\alpha{}' / k_\alpha = (\textstyle\sum_{i=1}^{s} \mathbf{W}_\alpha^i \mathbf{W}_\alpha^i{}') / [tr(\mathbf{W}_\alpha \mathbf{W}_\alpha{}') / n]$$
$$\mathbf{D}_g = \mathbf{W}_\delta \mathbf{W}_\delta{}' / k_\delta = (\textstyle\sum_{i=1}^{s} \mathbf{W}_\delta^i \mathbf{W}_\delta^i{}') / [tr(\mathbf{W}_\delta \mathbf{W}_\delta{}') / n]$$

The command for calculating genomic relationship matrices is:

```
./MNP -s grm -f geno -o test_sample_1
```

with the following output files,

```
test_sample_1_g.A
test_sample_1_g.D
```

These two SNP genomic relationship matrices are saved as binary files, and can be loaded for GBLUP and GREML runs to eliminate the calculations of the genomic matrix for every run.

8.3 MNP for haplotype additive genomic relationship matrix

8.3.1 Split haplotype files into smaller files

The first step splits each chromosome by user defined number of haplotype blocks. Assume two chromosomes 'hap_chr1.dat' and 'hap_chr2.dat' with 900 and 400 blocks respectively, the following splits each chromosome into 200 blocks:

```
./MNP -s split_big_file -i hap_chr1.dat -f hap -w 200 -o hap_sub_chr1
./MNP -s split_big_file -i hap_chr2.dat -f hap -w 200 -o hap_sub_chr2
```

This step will output the following smaller files:

```
hap_sub_chr1_1
hap_sub_chr1_2
hap_sub_chr1_3
hap_sub_chr1_4
hap_sub_chr1_5
hap_sub_chr2_1
hap_sub_chr2_2
```

In the above files, the first 4 files each has 200 blocks, the 5th has 100 blocks, and the 6th and 7th each has 200 blocks. These files are saved as text files.

8.3.2 Calculate $\mathbf{W}^{i}_{\alpha h}\mathbf{W}^{i}_{\alpha h}{}'$ for each split file

This step calculates $\mathbf{W}^{i}_{\alpha h}\mathbf{W}^{i}_{\alpha h}{}'$ for each of the split haplotype genotype files. The command is:

```
./MNP -s sub_ww -f hap -i hap_sub_chr1_1 -p 12
./MNP -s sub_ww -f hap -i hap_sub_chr1_2 -p 12
./MNP -s sub_ww -f hap -i hap_sub_chr1_3 -p 12
./MNP -s sub_ww -f hap -i hap_sub_chr1_4 -p 12
./MNP -s sub_ww -f hap -i hap_sub_chr1_5 -p 12
./MNP -s sub_ww -f hap -i hap_sub_chr2_1 -p 12
./MNP -s sub_ww -f hap -i hap_sub_chr2_2 -p 12
```

The $\mathbf{W}^{i}_{\alpha h}\mathbf{W}^{i}_{\alpha h}{}'$ matrices are saved in temporary folder 'tmp_haplo_a_ww_split_matrix' folder. This folder is temporary and is deleted after the calculations of the next step.

8.3.3 Calculate haplotype genomic relationship matrix and save as binary files

The MNP program will automatically use the saved $\mathbf{W}^{i}_{\alpha h}\mathbf{W}^{i}_{\alpha h}{}'$ matrices for calculation of the genomic relationship matrix using the following formula:

$$\mathbf{A}_{gh} = \mathbf{W}_{\alpha h}\mathbf{W}_{\alpha h}{}'/\,k_{\alpha h} = (\textstyle\sum_{i=1}^{s}\mathbf{W}^{i}_{\alpha h}\mathbf{W}^{i}_{\alpha h}{}')/[\mathrm{tr}(\mathbf{W}_{\alpha h}\mathbf{W}_{\alpha h}{}')/n]$$

The command for calculating genomic relationship matrices is:

```
./MNP -s grm -f hap -o test_sample_1
```

with the following output file,

```
test_sample_1_g.AH
```

This haplotype genomic relationship matrix is saved as binary files, and can be loaded for GBLUP and GREML runs to eliminate the calculations of the genomic matrix for every run.

9 Utility Programs

9.1 List of programs in the GVCHAP computing pipeline

Program	Description
**Data Preparation**	
**block-by-kb.py**	generate fixed kb distance block definition files
**block-by-snp.py**	generate fixed number of SNPs block definition files
**block-by-pos.py**	generate block definition files from a list of begin and end positions
**convert-from-vcf.py**	converts vcf to gvchap format
**convert-to-findhap.py**	converts gvchap format to findhap
**convert-to-vcf.py**	converts gvchap format to vcf
**count-haps.py**	counts haplotypes in chr file
**count-snps.py**	counts snps in chr file
**get-hap-geno.py**	creates haplotype genotype files based on block info files
**split-by-chr.py**	splits findhap genotypes.filled and haplotypes.txt output using chromosome.data by chromosomes
**GVCHAP Analysis**	
**GVCHAP**	Main program of GVCHAP analysis for GREML and GBLUP
**MNP**	Multi-node processing of haplotype and SNP genomic relationship matrices for large samples.
**Post-GVCHAP Analysis**	
**merge-mrk-hap.py**	merge the marker effects and heritabilities with haplotype heritabilities for visualization with SNPEVG2
**get-correlations.R**	calculate correlations of validation sample and original phenotype from gvcblup/gvchap output
**Other Utilities**	
**convert-plink-gvcblup.py**	converts plink format to gvcblup format
**convert-gvcblup-plink.py**	converts gvcblup format to plink format
**delete-rows.py**	deletes rows based on delete list in selected column
**ped-relationships.py**	calculate pedigree additive relationship matrix
**keep-rows.py**	keeps rows based on keep list in selected column
**stitch-hap.py**	generates haplotypes.txt from hap.list and hap.found output of findhap

9.2 Command line help information

The following is a list of help files with all possible options for each program. This information may also be accessed by running

```
program_name.extension -h
```

or

```
program_name.extension --help
```

Python 3.* is required for tools with *.py extension. R is required for get-correlations.R.

block-by-kb.py

```
usage: block-by-kb.py [-h] -i INPUT [-s SIZE] [-o OUTPUT] [-v]

generate x kb block definition file

optional arguments:
  -h, --help            show this help message and exit
  -i INPUT, --input INPUT
                        path to snp position file [required] format: 'snpid
                        chr pos'
  -s SIZE, --size SIZE  size in kbp (default: 500)
  -o OUTPUT, --output OUTPUT
                        output file name (default: hap_info)
  -V, --verbose         verbose output (default: False)
```

block-by-snp.py

```
usage: block-by-snp.py [-h] -i INPUT [INPUT ...] [--snp SNP] [-o OUTPUT]
                       [--nosort] [--noheader] [-V]

make x snp block definition file

optional arguments:
  -h, --help            show this help message and exit
  -i INPUT [INPUT ...], --input INPUT [INPUT ...]
                        path(s) to hap chr file(s) [required]
  --snp SNP             block size in snp (default: 4)
  -o OUTPUT, --output OUTPUT
                        output folder (default: hap_info)
  --nosort              set to not sort hap chr by number in filename
                        (default: True)
  --noheader            set if there is no header in the input files (default:
                        False)
  -V, --verbose         verbose output (default: False)
```

block-by-pos.py

```
usage: block-by-pos.py [-h] -p POS -m MAP [-o OUTPUT] [-V]

generate block definition files based on a list of positions for each block

optional arguments:
  -h, --help            show this help message and exit
  -p POS, --pos POS     path to block position file [required] format:
```

```
                        'chr:begin_pos:end_pos' without header
  -m MAP, --map MAP     path to map file [required] format: 'SNPID Chr Pos'
                        with header
  -o OUTPUT, --output OUTPUT

  --header              set if there is a header in the input block list (default:
                        False)
                        output file name (default: hap_info)
  -V, --verbose         verbose output (default: False)
```

convert-from-vcf.py

```
usage: convert-from-vcf.py [-h] -i INPUT [INPUT ...] [-m MISSING] [--map MAPFILE]
                   [--genofolder GENOFOLDER] [--hapfolder HAPFOLDER]
                   [--genoprefix GENOPREFIX] [--happrefix HAPPREFIX]
                   [--nosort] [--interval INTERVAL] [-V]

converts vcf to gvchap format

optional arguments:
  -h, --help            show this help message and exit
  -i INPUT [INPUT ...], --input INPUT [INPUT ...]
                        full path(s) to vcf chr file(s)
  -m MISSING, --missing MISSING
                        code for missing genotype in snp chr files (default:
                        -9999)
  --map MAPFILE         output path for map file formatted 'SNPID Chr
                        Position' (default: map_new.txt)
  --genofolder GENOFOLDER
                        output path for snp genotype chr files (default: geno)
  --hapfolder HAPFOLDER
                        output path for haplotype chr files (default: hap)
  --genoprefix GENOPREFIX
                        prefix for the seperated geno files (default: chr)
  --happrefix HAPPREFIX
                        prefix for the seperated hap files (default: chr)
  --nosort              do not sort vcf file by position (default: True)
  --interval INTERVAL   number of passes, reduces memory usage but is slower
                        (default: 1)
  -V, --verbose         verbose output (default: False)
```

convert-to-findhap.py

```
usage: convert-to-findhap [-h] -i INPUT [INPUT ...] -m MAP [--geno GENO]
                          [--chrdata CHRDATA] [--nosort] [-V]

gvchap format to findhap format

optional arguments:
  -h, --help            show this help message and exit
  -i INPUT [INPUT ...], --input INPUT [INPUT ...]
                        path(s) to snp chr file(s) [required]
  -m MAP, --map MAP     path to map file formatted 'SNP chr pos'
  --geno GENO           output path of genotypes.txt type file (default:
                        genotypes.txt)
  --chrdata CHRDATA     output path of chromosome.data type file (default:
                        chromosome.data)
  --nosort              do not sort input files by number in filename
```

```
                        (default: True)
  -V, --verbose         verbose output (default: False)
```

convert-to-vcf.py

```
usage: convert-to-vcf.py [-h] -i INPUT [INPUT ...] -m MAP [--ref REF] [--alt ALT]
                   [--qual QUAL] [--filter FILTER] [--info INFO]
                   [--format FORMAT] [-o OUTPUT] [--nosort] [-V]

converts findhap format to vcf

optional arguments:
  -h, --help            show this help message and exit
  -i INPUT [INPUT ...], --input INPUT [INPUT ...]
                        path(s) to snp chr file(s) [required]
  -m MAP, --map MAP     path to map file formatted 'SNP chr pos'
  --ref REF             reference allele (default: T)
  --alt ALT             alternative allele (default: C)
  --qual QUAL           VCF QUAL column (default: -)
  --filter FILTER       VCF FILTER column (default: -)
  --info INFO           VCF INFO column (default: -)
  --format FORMAT       VCF FORMAT column (default: GT)
  -o OUTPUT, --output OUTPUT
                        output name for vcf file (default: output)
  --nosort              do not sort input files by number in filename
                        (default: True)
  -V, --verbose         verbose output (default: False)
```

count-haps.py

```
usage: count-haps.py [-h] -i INPUT [INPUT ...] [-o OUTPUT] [-s]

counts haplotypes in chr file

optional arguments:
  -h, --help            show this help message and exit
  -i INPUT [INPUT ...], --input INPUT [INPUT ...]
                        path to input files [required]
  -o OUTPUT, --output OUTPUT
                        output file name (default: count-snps.log)
  --nosort              do not sort by number in filename (default: False)
```

count-snps.py

```
usage: count-snps.py [-h] -i INPUT [INPUT ...] [-o OUTPUT] [-p PREFIX] [-s]

counts snps in chr file

optional arguments:
  -h, --help            show this help message and exit
  -i INPUT [INPUT ...], --input INPUT [INPUT ...]
                        path to input files [required]
  -o OUTPUT, --output OUTPUT
                        output file name (default: count-snps.log)
  -p PREFIX, --prefix PREFIX
                        prefix in front of output lines (default: geno_snp)
  --nosort              do not sort by number in filename (default: False)
```

delete-rows.py

```
usage: delete-rows.py [-h] -i INPUT [INPUT ...] -c COLUMN -d DELETE [-V]
```

```
deletes rows based on delete list in selected column

optional arguments:
  -h, --help            show this help message and exit
  -i INPUT [INPUT ...], --input INPUT [INPUT ...]
                        input file
  -c COLUMN, --column COLUMN
                        column to search for delete values
  -d DELETE, --delete DELETE
                        file with list of delete values to search for
  -V, --verbose         verbose output (default: False)
```

get-hap-geno.py

```
usage: get-hap-geno.py [-h] --hap HAP [HAP ...] --hapinfo HAPINFO [HAPINFO ...]
                    [-m MISSING] [--missing-value MISSING_VALUE] [-o OUTPUT]
                    [-p OUTPUT_PREFIX] [--nosort] [--header] [-V]

creates haplotype genotype files based on block info files

optional arguments:
  -h, --help            show this help message and exit
  --hap HAP [HAP ...]   path(s) to hap chr file(s) [required]
  --hapinfo HAPINFO [HAPINFO ...]
                        path(s) to hap_info file(s) [required]
  -m MISSING, --missing MISSING
                        coding for missing alleles, blocks with the allele
                        will be set to missing-value (default: None)
  --missing-value MISSING_VALUE
                        value to use for blocks with missing alleles (default:
                        -9999)
  -o OUTPUT, --output OUTPUT
                        output folder for haplotype genotypes (default:
                        hap_geno)
  -p OUTPUT_PREFIX, --output-prefix OUTPUT_PREFIX
                        output prefix for haplotype genotype files (default:
                        hap_geno)
  --nosort              do not sort input files by number in filename
                        (default: False)
  --header              set if there is a header in the hap input files
                        (default: False)
  -V, --verbose         verbose output (default: False)
```

gvcblup-plink.py

```
usage: gvcblup-plink.py [-h] -p PHENOTYPE -g GENOTYPE [-fid FAMILY_ID]
                        [-id ID] [-pid PATERNAL_ID] [-mid MATERNAL_ID]
                        [-s SEX] [-t TRAIT] [-o OUTPUT] [-m MISSING_VALUE]
                        [-mp MAP] [-V]

converts gvcblup format to plink format

optional arguments:
  -h, --help            show this help message and exit
  -p PHENOTYPE, --phenotype PHENOTYPE
                        path to phenotype file [required]
  -g GENOTYPE, --genotype GENOTYPE
```

```
                        path to genotype file(s) [required]
  -fid FAMILY_ID, --family-id FAMILY_ID
                        family id column number (default: 0)
  -id ID, --id ID       individual id column number (default: 0)
  -pid PATERNAL_ID, --paternal-id PATERNAL_ID
                        paternal id column number (default: 0)
  -mid MATERNAL_ID, --maternal-id MATERNAL_ID
                        maternal id column number (default: 0)
  -s SEX, --sex SEX     sex of individual column number (default: 0)
  -t TRAIT, --trait TRAIT
                        single trait column number (default: 0)
  -o OUTPUT, --output OUTPUT
                        name of plink project (default: output)
  -m MISSING_VALUE, --missing-value MISSING_VALUE
                        missing value symbol (default: 9999)
  -mp MAP, --map MAP    missing value symbol (default: None)
  -V, --verbose         verbose output (default: False)
```

ped-relationships.py

```
usage: relationship.py [-h] -i INPUT [-o OUTPUT] [-id INDIVIDUAL_ID]
                       [-pid PATERNAL_ID] [-mid MATERNAL_ID] [-m MISSING]
                       [--header] [-V]

calculate pedigree relationship matrix based on the Tier algorithm (Tier, 1990) for
comparison with genomic additive relationships using SNPs

optional arguments:
  -h, --help            show this help message and exit
  -i INPUT, --input INPUT
                        path to input file [required]
  -o OUTPUT, --output OUTPUT
                        path to output file (default: output)
  -id INDIVIDUAL_ID, --individual-id INDIVIDUAL_ID
                        column of individual id (default: 1)
  -pid PATERNAL_ID, --paternal-id PATERNAL_ID
                        column of father's id (default: 2)
  -mid MATERNAL_ID, --maternal-id MATERNAL_ID
                        column of mother's id (default: 3)
  -m MISSING, --missing MISSING
                        missing value [default:0] (default: 0)
  --header              data contains heaader row (default: False)
  -V, --verbose         verbose output (default: False)
```

keep-rows.py

```
usage: keep-rows.py [-h] -i INPUT [INPUT ...] -c COLUMN -k KEEP [-s SUFFIX]
                    [--header] [-V]

keeps rows based on keep list in selected column

optional arguments:
  -h, --help            show this help message and exit
  -i INPUT [INPUT ...], --input INPUT [INPUT ...]
                        input file
  -c COLUMN, --column COLUMN
                        column to search for keep values
  -k KEEP, --keep KEEP  file with list of keep values to search for
```

```
  -s SUFFIX, --suffix SUFFIX
                        output suffix (added to end of file after .) (default:
                        new)
  --header              keep header (default: False)
  -V, --verbose         verbose output (default: False)
```

plink-gvcblup.py

```
usage: plink-gvcblup.py [-h] [--file FILE] [--ped PED] [--map MAP]
                        [-g OUTGENO] [-m OUTMAP] [-fid FAMILY_ID] [-id ID]
                        [-pid PATERNAL_ID] [-mid MATERNAL_ID] [-s SEX]
                        [-t TRAIT] [-o OUTPUT] [--missing-value MISSING_VALUE]
                        [--recode] [-V]

converts plink format to gvcblup format

optional arguments:
  -h, --help            show this help message and exit
  --file FILE, --plink FILE
                        plink project name
  --ped PED             path to ped file
  --map MAP             path to map file
  -g OUTGENO, --outgeno OUTGENO
                        path to output genotype file
  -m OUTMAP, --outmap OUTMAP
                        path to output map file
  -fid FAMILY_ID, --family-id FAMILY_ID
                        family id column number (default: 0)
  -id ID, --id ID       individual id column number (default: 0)
  -pid PATERNAL_ID, --paternal-id PATERNAL_ID
                        paternal id column number (default: 0)
  -mid MATERNAL_ID, --maternal-id MATERNAL_ID
                        maternal id column number (default: 0)
  -s SEX, --sex SEX     sex of individual column number (default: 0)
  -t TRAIT, --trait TRAIT
                        single trait column number (default: 0)
  -o OUTPUT, --output OUTPUT
                        name of chromosome files (default: chr)
  --missing-value MISSING_VALUE
                        missing value symbol (default: -9999)
  --recode              recode to 0,1,2 coding (default: False)
  -V, --verbose         verbose output (default: False)
```

split-by-chr.py

```
usage: split-by-chr.py [-h] [--hap HAP] [--chr CHR] [--geno GENO]
                       [--genofilled GENOFILLED] [--cutoff CUTOFF]
                       [--genofolder GENOFOLDER] [--hapfolder HAPFOLDER]
                       [--genoprefix GENOPREFIX] [--happrefix HAPPREFIX]
                       [-m MISSING_VALUE] [-V]

splits findhap genotypes.filled and haplotypes.txt output using
chromosome.data by chromosomes

optional arguments:
  -h, --help            show this help message and exit
  --hap HAP             path to haplotypes.txt type file (default: None)
  --chr CHR             path to chromosome.data type file (default:
                        chromosome.data)
```

```
  --geno GENO           path to genotypes.txt type file (default:
                        genotypes.txt)
  --genofilled GENOFILLED
                        path to genotypes.filled type file (default:
                        genotypes.filled)
  --cutoff CUTOFF       (int) minimum number of snps in original genotype.txt
                        (default: None)
  --genofolder GENOFOLDER
                        name for the seperated geno folder (default: geno)
  --hapfolder HAPFOLDER
                        name for the seperated hap folder (default: hap)
  --genoprefix GENOPREFIX
                        prefix for the seperated geno files (default: chr)
  --happrefix HAPPREFIX
                        prefix for the seperated hap files (default: chr)
  -m MISSING_VALUE, --missing-value MISSING_VALUE
                        missing value symbol (default: 9999)

  -V, --verbose         verbose output (default: False)
```

stitch-hap.py

```
usage: stitch-hap.py [-h] [--hap HAP] [--haplist HAPLIST]
                     [--hapfound HAPFOUND] [--chrdata CHRDATA] [--step STEP]
                     [-o OUTPUT] [-V]

generates haplotypes.txt from hap.list and hap.found output of findhap

optional arguments:
  -h, --help            show this help message and exit
  --hap HAP             path to haplotypes.txt type file (default: None)
  --haplist HAPLIST     path to hap.list type file (default: None)
  --hapfound HAPFOUND   path to hap.found type file (default: None)
  --chrdata CHRDATA     path to chromosome.data type file (default:
                        chromosome.data)
  --step STEP           which step number to use (default: 3)
  -o OUTPUT, --output OUTPUT
                        output file name (default: out_haplotypes.txt)
  -V, --verbose         verbose output (default: False)
```

mege-mrk-hap.py

```
usage: mege-mrk-hap.py [-h] -sh SNP_EFFECTS -hh HAP_HERITABILITIES -hi HAPINFO
                       [HAPINFO ...] --map MAP [-o OUTPUT] [--nosort] [-V]

merge the marker effects and heritabilities with haplotype heritabilities for
graphing with SNPEVG

optional arguments:
  -h, --help            show this help message and exit
  -sh SNP_EFFECTS, --snp_effects SNP_EFFECTS
                        path to SNP effects and heritabilites file from GVCHAP
                        [required]
  -hh HAP_HERITABILITIES, --hap_heritabilities HAP_HERITABILITIES
                        path to haplotype heritabilites file from GVCHAP
                        [required]
  -hi HAPINFO [HAPINFO ...], --hapinfo HAPINFO [HAPINFO ...]
                        path(s) to hap_info file(s) [required]
  --map MAP             path to map file (default: map.txt)
```

```
  -o OUTPUT, --output OUTPUT
                        output folder (default: combined.snpe)
  --nosort              set to not sort hap chr by number in filename
                        (default: True)
  -V, --verbose         verbose output (default: False)
```

get-correlations.R

```
usage: get-correlations.R [options]

calculates correlations of validation individuals and original phenotype from
gvcblup/gvchap output

optional arguments:
        -p CHARACTER, --phen=CHARACTER
                name of the phenotype file

        -g CHARACTER, --gblup=CHARACTER
                name of gblup file

        -i CHARACTER, --id=CHARACTER
                name of id column in phenotype file

        -t CHARACTER, --trait=CHARACTER
                name of original trait in phenotype file

        -v CHARACTER, --validation=CHARACTER
                name of validation sample

        -b CHARACTER, --blup=CHARACTER
                name(s) of blup column to correlate with in gblup file separated by
commas, no spaces

        -m INTEGER, --missing=INTEGER
                number to be used as missing value [default=-9999]

        -o CHARACTER, --output=CHARACTER
                name of the output file [default=out.txt]

        -V, --verbose
                print verbose output [default=FALSE]

        -S CHARACTER, --skip=CHARACTER
                number of lines to skip in gblup file [default=0]

        -h, --help

                show this help message and exit.
```

Author Contributions

YD conceived this study. DP is the author of the current version of GVCHAP and the utility programs. CW is the author of the initial version of GVCHAP. ZL is the author of the MNP program. CB and CT provided extensive evaluation that improved GVCHAP and the utility programs. ZL and DP evaluated computing time required by GVCHAP. DP, ZL and YD prepared the user manual.

Acknowledgements

This research was supported by USDA National Institute of Food and Agriculture Grant no. 2011-67015-30333 and 2018-67015-28128, and by project MN-16-124 of the Agricultural Experiment Station at the University of Minnesota. Supercomputer computing time was provided by the Minnesota Supercomputer Institute at the University of Minnesota.

References

Browning, B. L., Y. Zhou, and S. R. Browning. 2018. A one-penny imputed genome from next-generation reference panels. The American Journal of Human Genetics 103(3):338-348.

Da, Y. 2015. Multi-allelic haplotype model based on genetic partition for genomic prediction and variance component estimation using SNP markers. BMC Genetics 16(1):144.

Prakapenka, D., C. Wang, Z. Liang, C. Bian, C. Tan, and Y. Da. 2020. GVCHAP: A Computer Package for Genomic Prediction and Estimation Using Haplotypes and Single SNPs. Frontiers in Genetics, 11:282. doi: 10.3389/fgene.2020.0028.

Tier, B. 1990. Computing inbreeding coefficients quickly. Genetics Selection Evolution 22(4):419.

VanRaden, P. M., Sun. C., O'Connell, J. R. (2015). Fast imputation using medium or low-coverage sequence data. BMC genetics 16(1):82.

Wang, C., D. Prakapenka, S. Wang, S. Pulugurta, H. B. Runesha, and Y. Da. 2014. GVCBLUP: a computer package for genomic prediction and variance component estimation of additive and dominance effects. BMC Bioinformatics 15(1):270.
